# Bystander training to prevent gender-based violence against female university students: a scoping review*

**DOI:** 10.1590/1980-220X-REEUSP-2025-00231en

**Published:** 2026-08-03

**Authors:** Taís Tasqueto Tassinari, Letícia de Mello Padoin, Laís Alves Molina, Cristiane Cardoso de Paula, Stela Maris de Mello Padoin

**Affiliations:** 1 Prefeitura Municipal de Penha, Departamento de Vigilância Sanitária, Penha, SC, Brazil.; 2 Universidade Federal de Santa Catarina, Programa de Pós-graduação em Serviço Social, Florianópolis, SC, Brazil.; 3 Universidade Franciscana, Residência Profissional em Enfermagem Obstétrica, Santa Maria, RS, Brazil.; 4 Universidade Federal de Santa Maria, Programa de Pós-graduação em Enfermagem, Santa Maria, RS, Brazil.

**Keywords:** Prevention, Universities, Gender-Based Violence, Scoping Review, Women.

## Abstract

**Objective:**

To map the characteristics of bystander training to prevent gender-based violence against female students in a university context.

**Method:**

Scoping review using the JBI method. The search was conducted in March 2025 in MEDLINE/PubMed, WoS, BVS, EBSCO, EMBASE, Scopus, PsycInfo/APA, and SciELO, regardless of language, with a time frame of 2016. The selection and extraction were double-independent, with descriptive analysis of the characterization and inductive approach to the content of the training.

**Results:**

Nine types of training were identified in 46 included studies that aimed to prevent sexual violence using a mixed-gender approach. The training content was categorized into: definitions, concepts, and epidemiology of violence; rape myths and gender stereotypes; consent and healthy communication; attitudes, behaviors, and interventions of bystanders; available resources and services; values, awareness, and personal ethics; risks, barriers, and obstacles to intervention.

**Conclusion:**

The gap lies in the thematic concentration of studies, focused on sexual violence, with limited exploration of risks, barriers to intervention, and institutional resources in the content of the strategies.

## INTRODUCTION

Gender-based violence is a global problem observed in social and economic contexts rooted in unequal power relations, with a patriarchal structure, primarily affecting women^(1)^. They are the most affected in the university context, and the prevalence of sexual violence among students may be higher than in the general population^(2)^. A review study of university students found prevalence rates ranging from 1.3% to 88%, with wide variation depending on the type of violence, the data collection tool, and the study sample^(3)^.

Gender-based violence among female university students manifests in various forms; perpetrators include current and former partners, classmates and other students, faculty, and university staff^(4)^. The violence is often normalized within their daily lives, occurring in domestic environments (such as their own homes and those of the perpetrators), academic spaces (such as classrooms and on-campus common areas) and parties^(5)^.

Violence againstfemale university students severely impacts their physical health, mental well-being, and academic performance. Intimate partner violence can induce depression and post-traumatic stress disorder, the severity of which is significantly correlated with the intensity of the violence^(6)^. In addition to symptoms of anxiety and suicidal behavior associated with experienced violence, findings from a systematic review showed that sexual violence was associated with lower grades, absenteeism, and school dropout rates^(7)^.

The normalization and trivialization of violence in educational settings exacerbates the situation for women experiencing it. Frequently, experiences of gender-based violence are silenced and victims’ accounts are invalidated to protect the institution’s reputation^(8)^. The process of “institutional betrayal” is rooted in social structures of power and oppression, contributing to the perpetuation of violence. It manifests across various institutional levels through policies that hinder reports of violence, impose bureaucratic procedures, and fail to remove high-ranking perpetrators, alongside a lack of engagement to promote systemic and lasting change in universities^(9)^.

To better understand the possibilities of confronting gender-based violence in the university environment, it is relevant to map preventive strategies that promote social engagement for accountability and cultural transformation of gender roles. To this end, the literature points to interventions involving bystander training, which rest on the assumption that community leaders can foster social change to tackle public health issues. The term ‘bystander’ refers to individuals who witnessed a violent event before, during, and/or after its occurrence. Bystander training thus aims to empower individuals to intervene on acts of violence or social norms that perpetuate violence, working toward prevention^(10)^.

Several factors can promote effective bystander behavior, contributing to violence prevention. Some examples include educational content and messages that help bystanders identify situations of violence; skill-building and practice to increase the bystander’s sense of efficacy through replicable intervention models; and intervention approaches that respect and consider the bystander’s safety so that the benefits of intervening outweigh the barriers^(11)^.

Hence, bystander training strengthens the skills necessary to reduce violence risks by enabling individuals to recognize potentially violent situations, intervene safely and effectively, and confront attitudes that encourage or tolerate violent behavior. These interventions aim to mitigate violence by shifting social norms and transforming bystander attitudes, beliefs, and intentions to act. By disrupting passivity in the face of violence, this approach can reshape and encourage shared social responsibility, preventing gender-based violence^(12)^.

The literature features numerous studies on bystander training, indicating different intervention formats^(13,14,15)^. However, efforts to understand and systematize prevention strategies are still fragmented. Existing data are scattered regarding methods and core characteristics, such as intervention duration, content, delivery format, and application context. Identifying this gap underscores the necessity of mapping the available evidence on training programs designed to prevent gender-based violence.

Furthermore, a preliminary search was conducted, and no ongoing or completed scoping reviews or protocols were identified in the International Prospective Register of Systematic Reviews (PROSPERO), the Open Science Framework (OSF), the Medical Literature Analysis and Retrieval System Online (MEDLINE), the Cochrane Database of Systematic Reviews, or JBI Evidence Synthesis. This confirms the knowledge gap on the subject and the need to develop a scoping review. Therefore, the objective of this review was to map the characteristics of bystander training programs designed to prevent gender-based violence against female students in university contexts.

## METHOD

### Study Design

This is a scoping review conducted in accordance with the JBI methodology and reported following the Preferred Reporting Items for Systematic Reviews and Meta-Analyses extension for Scoping Reviews (PRISMA-ScR) guidelines^(11,12)^. The protocol was registered on the Open Science Framework (https://doi.org/10.17605/OSF.IO/4NK9U).

### Identifying the Review Question

The review question (“What are the characteristics of bystander training programs to prevent gender-based violence against female students in university contexts?”) was structured using the PCC acronym: Population (**P**: university students), Concept (**C**: prevention of gender-based violence among female university students through bystander training), and Context (**C**: university campuses).

Based on the primary research question, the following sub- questions were developed: What types of interventions are described in the literature? How will the interventions be delivered? Do the interventions involve a facilitator? How long do the interventions last? How many sessions or stages are there in the interventions? What is the content of the interventions?

### Eligibility Criteria

Participants: Eligible studies were those conducted with university students (regardless of sex, gender, or age) enrolled in undergraduate or graduate programs. The review included both general and specific student populations, including female-only samples, undergraduate or postgraduate cohorts, and members of sororities and fraternities.

Concept: Studies were eligible if they described bystander training interventions aimed at preventing gender-based violence against female university students (inclusive of cisgender and transgender women), encompassing intimate partner violence such as physical, sexual, psychological, emotional, or economic abuse. Eligible interventions could be delivered in various formats (e.g., educational programs, videos, online modules, campaigns, workshops, or lectures), with no restrictions on duration or number of sessions, and could be either facilitator-led or self-administered. Conversely, studies were excluded if they focused on professional strategies for violence prevention, self- defense, and those combining violence prevention with the prevention of Sexually Transmitted Infections (STIs) or alcohol abuse, due to the complexity of synthesizing these themes.

Context: Studies conducted on the campuses of public or private universities in any country, as well as those delivered via digital platforms, were eligible. Interventions carried out outside of higher education environments were excluded, even if institutionally supported.

Study design: Eligible studies included those with experimental and quasi-experimental designs, including controlled before-and-after studies and interrupted time series, as well as observational, qualitative, and mixed-methods research. Following an exploratory search, grey literature was found to have limitations regarding methodological descriptions and detailed accounts of the training programs, which would compromise the consistency in clarity, selection, and completeness required for data extraction. Consequently, editorials, opinion pieces, reflective essays, epidemiological bulletins, state and federal legislation, guidelines, primers, and technical notes were excluded.

Regarding the publication window, a temporal marker was established based on the International Day of Women and Girls in Science, on February 11, approved by the United Nations General Assembly on December 22, 2015. Although this milestone is symbolic and political, its adoption as a methodological criterion is justified as it marks a global commitment to a paradigm shift to ensure gender equality in research environments. Consequently, studies published from 2016 onward were eligible for this review. This timeframe successfully aligns the emerging paradigm of protecting female students in academic settings with a bibliographic survey of practical interventions, as well as with an era of institutional mobilization to address violence in teaching and research contexts.

### Search Strategy and Information Sources

Following the JBI methodological recommendations, the search strategy was carried out in three stages. Stage 1 consisted of a search limited to the MEDLINE/PubMed database to analyze the keywords and index terms contained the titles and abstracts of the identified articles. Following this initial mapping, search strategies were developed for other information sources (Stage 2).

The information sources consulted to retrieve studies comprised 8 virtual databases and libraries: MEDLINE/PubMed, Web of Science Core Collection (WoS), the Virtual Health Library (VHL), EBSCO, EMBASE, Scopus; APA PsycInfo, and the Scientific Electronic Library Online (SciELO). Studies published in any language were included. All searches were conducted in March 2023 and updated in February-March 2025. Stage 3 consisted of screening the reference lists of the included articles, but no additional studies were identified. All stages were carried out in collaboration with a specialized health sciences review librarian.

### Selection of Studies

The retrieved references were grouped and imported into Endnote 20/2020 (Clarivate Analytics, PA, USA). Following duplicate removal, the citations were exported to the Rayyan platform (Qatar Computing Research Institute, QCRI)^(16)^. Two reviewers independently screened titles and abstracts in a double-blind manner against the eligibility criteria. Potentially relevant studies were retrieved in full text and independently assessed for eligibility. The reasons for excluding full-text studies were documented and reported. At each stage of the selection process, any disagreements between reviewers were resolved through discussion with a third reviewer, who is an expert on the topic of gender-based violence. The search results and study selection process are fully presented in a PRISMA flow diagram^(17)^.

### Data Extraction and Presentation of Results

Data extraction was conducted in multiple phases. For the characterization phase, a standardized tool was used to map study characteristics, including country, year, journal, population, objective, and methodology^(18)^. For the concept mapping phase, the following data were extracted: type of intervention (poster presentation, video, online course, educational game, etc.), intervention content, intervention characteristics (duration, presence or absence of a facilitator, number of sessions and/or stages, specific type of violence it aims to prevent), and delivery method (in-person or online). Notably, seven studies evaluated two different interventions, while certain identical interventions were examined across different studies. Consequently, the total number of included studies differs from the total number of strategies mapped.

### Data Analysis

Descriptive analysis was used to characterize the primary studies and the attributes of the training programs, using simple frequency. These findings are synthesized and presented through tables and figures alongside descriptive text. To analyze the training content, an inductive approach to basic qualitative content analysis was used. This descriptive method involves open coding to allocate features into general categories^(19)^, executed in three phases: 1) Preparation: immersive reading of the sources of evidence to achieve data familiarization, focusing on identifying the core content of bystander training strategies designed to prevent gender-based violence against female students in university contexts; 2) Organization: identification of the topics of each strategy and the open coding of content into categories based on thematic similarity; 3) Reporting: descriptive and graphical presentation of the results^(20)^.

The methodological quality assessment was not performed, considering the distinction between systematic and scoping reviews, where the former should include a critical evaluation or risk of bias assessment to recommend the synthesized results for implementation in policies or practices. In contrast, scoping reviews are not required to assess the quality of the evidence included, but rather to provide an overview of the knowledge landscape to recommend the description of the mapped evidence and identify existing research gaps^(18)^.

## RESULTS

**Figure 1:**
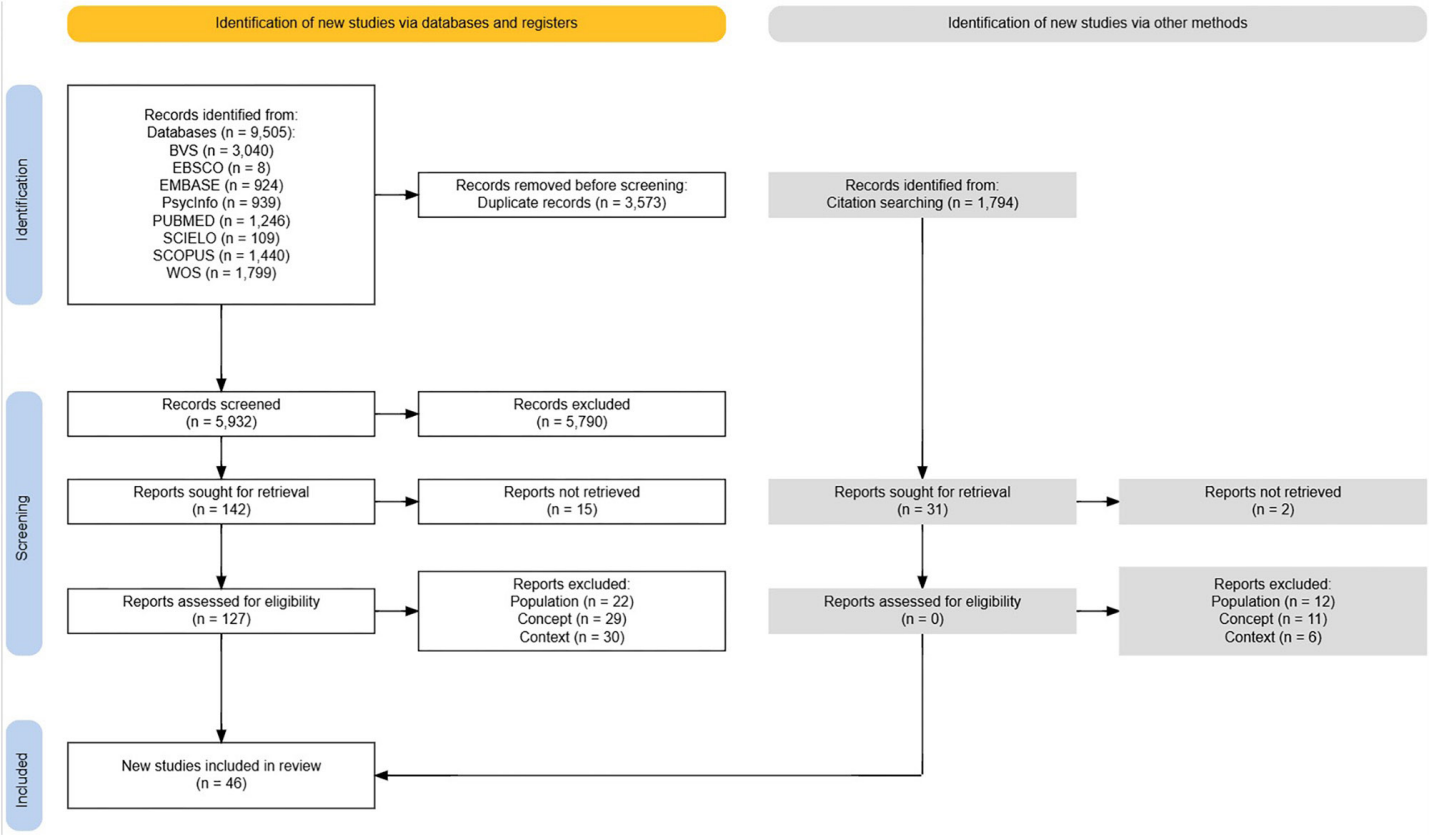
Flowchart Preferred Reporting Items for Systematic Reviews and Meta-Analyses extension for Scoping Review (PRISMA-ScR – Santa Maria, RS, Brazil, 2025).

The retrieved references were compiled and imported into Endnote 20/2020 reference management software. After duplicate removal, the remaining citations were exported to the Rayyan platform for title and abstract screening. Based on the eligibility criteria, a total of 127 studies advanced to full- text review. Of these, 81 were excluded for failing to meet the eligibility criteria, leaving 46 studies to comprise the final body of this review (Figure 1).

The country of origin, publication year, objective, study design, sample size, and participant gender for each included study are shown in Chart 1. In the primary studies, gender reporting lacked uniformity. Therefore, data extraction adhered to the terminology used within each study. In Chart 1, this information has been summarized and represented using symbols, specifically for illustrative and organizational purposes.

**Chart 1:** Characterization of the included studies – Santa Maria, RS, Brazil, 2025.

Country and year	Objective	Type of study and participants
USA, 2017^(21)^	To investigate the effectiveness of The Women’s Program, a sexual assault awareness program geared toward women.	Quasi-experimental. n:103 ♀
USA, 2018^(22)^	To examine how two methods of prevention delivery worked separately and together to promote attitude change related to sexual violence among college students.	Quasi-experimental. n:948 ♀ ♂
USA, 2019^(23)^	To provide a description and evaluation data from a sexual assault prevention program administered to first-year students during their first semester at a large public university.	Quasi-experimental. n:2,305 ♀ ♂
USA, 2016^(24)^	To examine whether a brief and easy-to-implement university-wide intervention could increase bystander behaviors, intentions, attitudes, efficacy, and social norms to prevent dating violence; and to understand what variables were most strongly associated with bystander behaviors.	Quasi-experimental. n:288 ♀ ♂
USA, 2021^(25)^	To explore the efficacy of an intervention called “bystander plus” programming.	Quasi-experimental. n:81 ♀ ♂
USA, 2022^(13)^	To describe the development and evaluation of a widely disseminated sexual and dating violence primary prevention program (Red Flag Campaign).	Quasi-experimental. n:203 ♀ ♂
USA, 2016^(26)^	To examine the effects of Green Dot on rates of violence victimization and perpetration among first-year students with data collected over a 4-year period (2010–2013).	Quasi-experimental. n:7,111 ♀ ♂
USA, 2018^(27)^	To assess the extent to which the workshops Violence Against Women Prevention Program decrease students’ rape myth acceptance, increase their knowledge and understanding of the University’s affirmative consent policy, and improve their confidence in interpreting cues related to sexual consent.	Experimental. n:1,957 ♀ ♂
Australia 2021^(28)^	To increase awareness and knowledge of pre-registration health students on the context of gender-based violence and for participants to learn bystander approaches to effectively intervene and reduce violence.	Quasi-experimental. Pilot study. n:21♀ ♂
USA, 2021^(29)^	To evaluate Wingman 101, a bystander-based sexual violence prevention program designed for male undergraduate athletes and fraternity members.	Quasi-experimental. n:80 ♂
USA, 2020^(14)^	To examine the effectiveness of Safe Sisters, a sexual assault bystander intervention program that targets members of college sororities.	Experimental. n:139 ♀
USA, 2017^(30)^	To identify predictors of bystander behaviors and sexual assertiveness among sorority women. To evaluate changes in rape myth acceptance, bystander self-efficacy, and sexual assertiveness two weeks after The Women’s Program was provided to sorority members, and to report bystander behaviors for two weeks after the intervention.	Quasi-experimental. n:141♀
Australia 2024^(15)^	To evaluate an online sexual violence prevention and response education module designed for and implemented in one Australian university.	Mixed. n:113♀ ♂O
USA, 2018^(31)^	To examine the effectiveness of a modified Green Dot bystander intervention training for resident assistants.	Quasi-experimental. n:24♀ ♂
USA, 2017^(32)^	To examine prevention programming focused on multiple aspects of sexual assault, including content on sexual consent, bystander intervention, reporting of sexual assault incidents, seeking help after an incident, and the support of sexual assault victims.	Quasi-experimental. n:146♀ ♂
USA, 2018^(33)^	To adapt, replicate, and test the generalization of the program Bringing in the BystanderTM at a private Jesuit Catholic liberal arts college in Massachusetts.	Quasi-experimental. n:164♀ ♂
USA, 2021^(34)^	To evaluate whether a semester-long course for first-year undergraduates influenced knowledge, attitudes, and behavioral intentions about gender, sexuality, and sexual violence.	Quasi-experimental. n:109♀ ♂
USA, 2016^(35)^	The present research reports on two randomized, controlled trials evaluating TakeCARE, a video bystander program designed to help prevent sexual violence on college campuses.	Experimental. Study 1: n:213 ♀ ♂ Study 2: n:211 ♀ ♂
USA, 2016^(36)^	To present an evaluation of a new procedure designed to measure bystander behavior to prevent sexual violence.	Quasi-experimental. n:91♀ ♂
USA, 2020^(37)^	To replicate and extend previous findings of TakeCARE’s effects on bystander behavior, testing two methods of video administration and examining moderators of TakeCARE’s effects on bystander behavior.	Experimental. n:557 ♀ ♂
Australia 2020^(38)^	To provide an overview of the implementation process, challenges and successes encountered at an Australian university in adopting the evidence based accredited workshop Sex, Safety and Respect.	Experience Report. n:118♀ ♂
USA, 2020^(39)^	To describe a bystander intervention campaign implemented at a Historically Black College or University in the Southeastern United States.	Qualitative. n:406♀ ♂
USA, 2021^(40)^	To evaluate the effectiveness a 5-year social norms sexual violence prevention marketing campaign designed specifically for men on a large public university in the Southeastern United States.	Time series. n:4,158 ♂
India 2022^(41)^	To evaluate the effectiveness of RISE, a sexual violence prevention program for female college students in India.	Quasi-experimental. n:245 ♀
USA, 2021^(42)^	To assess the efficacy of STOP Dating Violence, an online intervention developed to educate students about dating violence and appropriate bystander interventions on college campuses.	Experimental. n:317♀ ♂
USA, 2018^(43)^	To compare the effectiveness of a 90-min bystander education program for dating violence prevention with a traditional dating violence awareness education program, as well as to a no education control group, in changing attitudes, beliefs, perceived efficacy, intentions, and self-reported behaviors in college students.	Quasi-experimental. n:1,001♀ ♂
USA, 2019^(44)^	To document the creation of two video games to teach college students bystander intervention skills in situations of sexual and relationship violence and stalking.	Quasi-experimental. n:305 ♀ ♂
USA, 2021^(45)^	To evaluate the efficacy of two video games aimed at university students: (1) a trivia game and (2) an adventure game to train students on bystander intervention skills to prevent sexual violence.	Experimental. n:227 ♀ ♂
USA, 2018^(46)^	To test the efficacy of the Escalation workshop to prevent dating abuse with a sample of undergraduate students.	Experimental. n:85♀ ♂
USA, 2018^(47)^	To examine the process by which a web-based sexual violence prevention program (RealConsent) prevents sexual violence and improves bystander behavior.	Experimental. n:743♂
New Zealand 2021^(48)^	To present the quantitative and qualitative findings from a process of piloting the Bring in The Bystander Programme, developed to tackle the problem of sexual violence on university campuses by taking a community values approach.	Mixed. QUAN+qual. Pilot study. n:167♀ ♂
USA, 2018^(49)^	To present the implementation and evaluation of a theory-based campaign to promote active bystander intervention.	Quasi-experimental. n:1,505♀ ♂
USA, 2016^(50)^	To assess the recall of, reaction to, and understanding of a brief campus banner campaign promoting consent in sexual relationships, and to determine whether campaign exposure was associated with subsequent engagement in activities related to sexual assault education, awareness, and prevention.	Mixed. n:628 ♀ ♂
China 2019^(51)^	To evaluate the feasibility of Dating Compassion, Assessment, reFerral, and Education (CAFE) Ambassador Programme in China.	Quasi-experimental. n:85 ♀ ♂
USA, 2022^(52)^	To evaluate a brief, online intervention to reduce sexual aggression perpetration and increase prosocial bystander behaviors among heterosexual male college students.	Quasi-experimental. n:241♂
Vietnam 2022^(53)^	To adapt RealConsent, a theoretically grounded, web-based sexual violence prevention program to be suitable for university men in Vietnam, and to test the impact of the adapted program, GlobalConsent, in this setting.	Experimental. n:730♂
Vietnam 2023^(54)^	To test the impact of GlobalConsent in sexually violent behavior and prosocial bystander behavior among university men in Vietnam.	Experimental. n:793♂
USA, 2021^(55)^	To examine the short-term impact of a widely utilized sexual violence prevention course for matriculating college students as a population-level prevention approach.	Quasi-experimental. n:167.424 ♀ ♂TO
England 2025^(56)^	To explore the feasibility of implementing the “The Intervention Initiative” in a university setting in the north of England.	Mixed. n:21 ♀ ♂ (FG and interviews)
USA, 2023^(57)^	To evaluate the effectiveness of an interactive, personalized safety decision and planning tool, myPlan app, for concerned friends (university students of any gender aged 18–24) who self-identify as having a female friend who has experienced intimate partner violence in the last 6 months.	Experimental. n:293 ♀ ♂
USA, 2024^(58)^	To examine the effects of an awareness intervention on bystander behavior among university students who completed a bystander training program.	Mixed. Study 1: n:13♀ ♂NB Study 2: n:38 ♀ ♂NB
Canada 2023^(59)^	Using meta-analytic techniques to explore data from an in-person program to prevent relationship violence (delivered before COVID-19).	Quasi-experimental. Time series. n:244 ♀ ♂NB,T
USA, 2024^(60)^	To examine the impact of a self-paced online program focusing on bystander intervention for domestic violence.	Quasi-experimental. n:293 ♀ ♂
USA, 2023^(61)^	To describe the development of the SPoRT prevention intervention and its initial evaluation of feasibility and acceptability.	Mixed. n:30 ♀ ♂
India 2024^(62)^	To investigate the short-term effects of the RISE-ON modules on participants’ knowledge and attitudes.	Quasi-experimental. n:244♀
USA, 2023^(63)^	To develop and conduct a preliminary assessment of Make a Change, a new digital application designed to prevent sexual violence among 2nd- to 4th-year college students.	Mixed. n:105♀ ♂

Legend: ♀ women;

♂ men;

NB: non-binary; T: transgender; O: other genders; FG: focus groups.

Regarding the countries of origin, the studies were predominantly conducted in the United States of America (n = 35), followed by Australia (n = 3), India (n = 2), Vietnam (n = 2), New Zealand (n = 1), China (n = 1), Canada (n = 1), and England (n = 1). Of the 46 studies included, 34 presented strategies aimed at mixed-gender groups, 7 focused exclusively on men, and 5 on women.

Regarding **specific types of violence** addressed, the studies aimed to prevent sexual violence (34), domestic and intimate partner violence (14), and interpersonal or gender-based violence against women (addressed individually or concurrently; 4).

**Figure 2:**
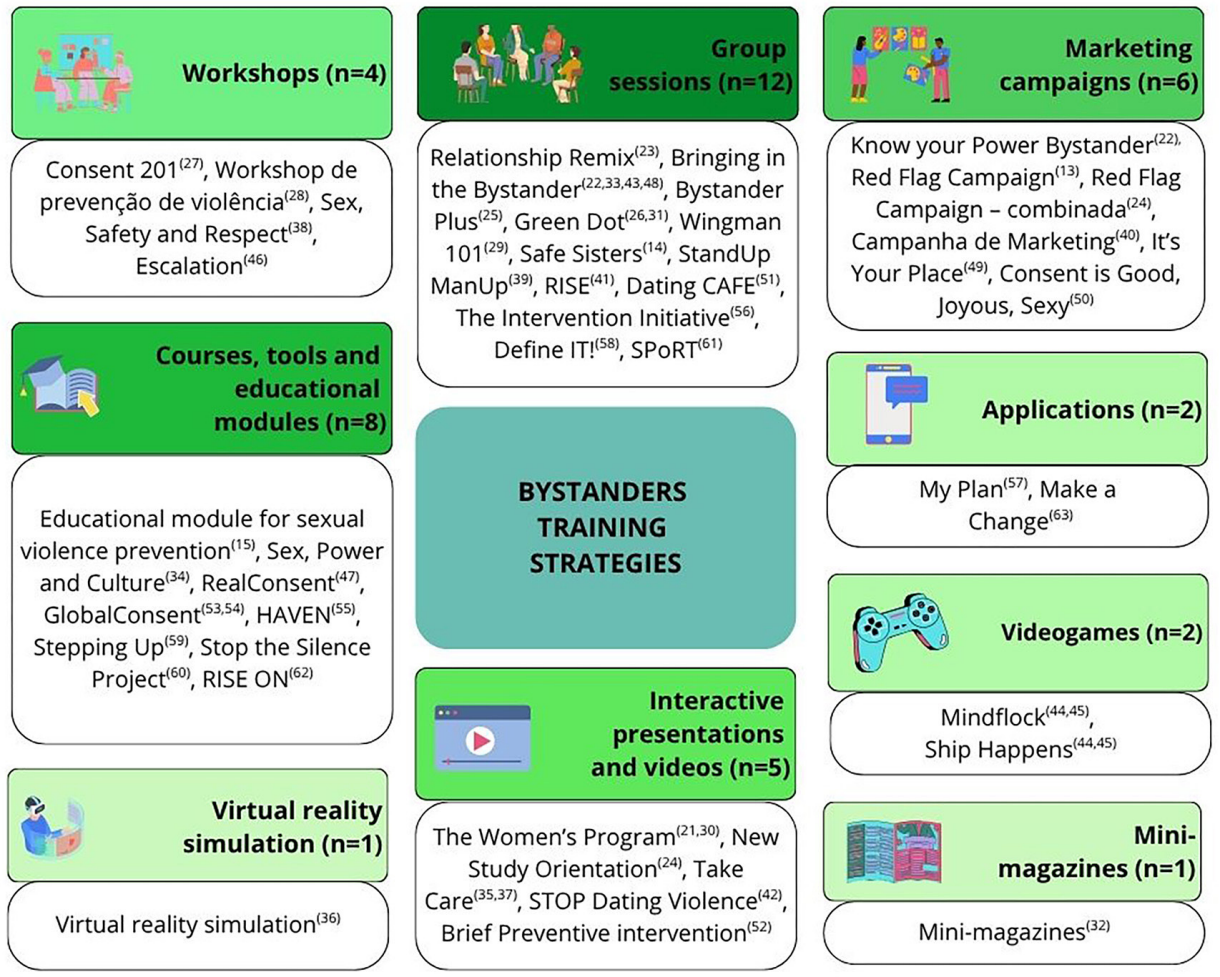
Identification of strategies to prevent gender-based violence, classified by type – Santa Maria, RS, Brazil, 2025.

Across the 46 included studies, 41 different training strategies were identified. In some cases, the same study presented evidence for two different interventions, and some interventions appeared in more than one publication. These **strategies were classified into nine types**: group sessions (12)^(14,22,23,25,26,29,31,33,39,41,43,48,51,56,58,61)^, interactive presentations and videos (5)^(21,24,30,35,37,42,52)^, marketing campaigns (6)^(13,22,24,40,49,50)^, courses, modules, and educational tools (8)^(15,34,47,53,54,55,59,60,62)^, workshops (4)^(27,28,38,46)^, video games (2)^(44,45)^, digital applications (2)^(57,63)^, virtual reality simulation (1)^(36)^, and mini-magazine (1)^(32)^ (Figure 2).

Regarding the delivery methods **of the 41 strategies,** they were classified into in-person (25)^(14,21,22,23,24,25,26,27,28,29,30,31,32,33,34,38,39,40,41,43,46,48,50,51,56,58,59,61)^, digital or online (11)^(15,42,44,45,47,53,54,55,57,60,62,63)^, and hybrid formats, the latter combining face-to-face actions with digital elements (5)^(13,35,36,37,49,52)^.

The **presence of facilitators** was specifically identified in in-person strategies, including group sessions (11)^(14,22,23,25,26,29,31,33,39,41,43,48,51,56,58,61)^, workshops (4)^(27,28,38,46)^,interactive presentations and videos (2)^(21,24,30)^, and educational courses, modules, or tools (2)^(34,59)^. In total, 20 strategies reported utilizing facilitators.

The **duration and frequency of the interventions** varied across the different types of strategy. Group sessions ranged from 1 to 8 hours, delivered either as a single session^(14,23,31,33,43,56,58)^ or distributed across two^(25,30,48)^, three^(51)^, or four sessions^(61)^, with frequency remaining unreported in two studies^(26,41)^. All workshops consisted of single sessions lasting from 40 minutes up to a half-day block^(27,28,38,46)^. Videos and interactive presentations were designed as one-time exposures ranging from 24 to 90 minutes^(21,24,30,35,37,42,52)^. In contrast, marketing campaigns were deployed over highly variable periods (from one week to five years), facilitating repeated exposure^(13,22,24,40,49,50)^. The educational courses, modules, and tools ranged in length from 45 minutes to a full academic semester, formatted as either a single presentation^(15,55,62)^ or divided into modules^(34,47,53,54,59)^ (data were unavailable for one study^(60)^). For digital and print formats, video games lasted 45 to 60 minutes^(44,45)^; mini-magazines comprised five 8-page volumes delivered by mail^(32)^; virtual reality programs involved seven different simulations lasting 2 to 4 minutes each^(36)^; and both mobile applications offered a 30-minute core duration^(63)^ and unlimited user access for one year^(57)^.

**Figure 3:**
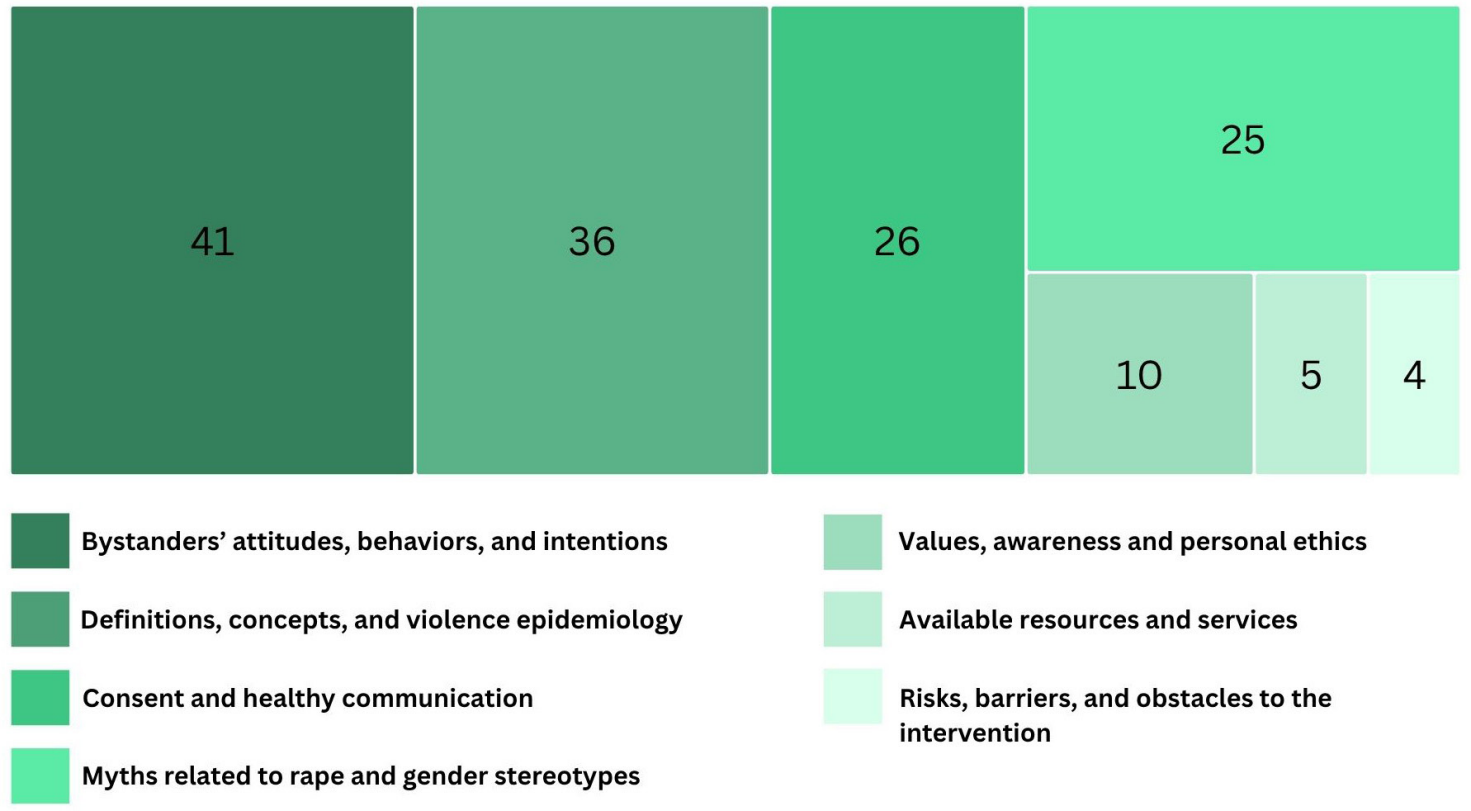
Quantitative distribution of identified studies by content category – Santa Maria, RS, Brazil, 2025.

Regarding the **content of the strategies**, seven thematic categories were identified: 1. Definitions, concepts, and epidemiology of violence (n = 36); 2. Rape myths and gender stereotypes (n = 25); 3. Consent and healthy communication (n = 26); 4. Bystander attitudes, behaviors, and intentions (n = 41); 5. Available resources and services (n = 5); 6. Values, awareness, and personal ethics (n = 10); and 7. Risks, barriers and obstacles to intervention (n = 4) (Figure 3).

## DISCUSSION

Regarding **geographic origin**, the United States predominated, representing 76% of the analyzed sample. These findings are consistent with previous systematic reviews on sexual assault prevalence and gender-based violence interventions, which reported that approximately 73% of their primary studies originated from the USA^(64,65)^. This concentration can be understood as a result of US federal policy frameworks, such as Title IX, which requires federally funded institutions to implement violence prevention strategies while prohibiting sex-based discrimination in educational programs and activities^(66)^. Given the high volume of studies, it can be inferred that the existence of legislative mandates encourages data production on violence prevalence in universities, as well as the development of prevention strategies.

Regarding study participants, there was a **predominance of strategies aimed at mixed-gender groups**. In this regard, a systematic review and meta-analysis evaluated the effectiveness of bystander programs addressing sexual violence on college campuses and found no significant differences in efficacy between mixed-gender and same-sex groups. These effects were examined across two outcomes: bystander attitudes and/or beliefs, and behaviors^(67)^.

Strategies targeting mixed-gender groups also align with student expectations, according to findings from a study on preferences regarding on-campus violence prevention. Students perceive mixed-gender programs as more inclusive of gender and sexual diversity, as well as effective at dispelling stereotypes about the roles men and women play in sexual assault incidents^(68)^.

Within this review, six articles present prevention strategies aimed solely at male groups. Evidence from a systematic review indicates that prevention programs targeting men and boys show promising results in shifting attitudes, beliefs, and social norms related to violence^(69)^. One hypothesis raised in the literature is that men are more receptive to and comfortable with bystander training content and feel less judged by their peers when participating in single-sex groups^(70)^. It is important to address the social construction of masculinity across various social institutions, which reinforces patriarchal power dynamics of men over women. Confronting these dynamics requires the renunciation of gender-based privileges attributed to men; consequently, men may perceive the restructuring of these power systems as a personal loss^(71)^.

Therefore, planning strategies to increase male engagement in gender-based violence prevention programs in universities is critical, as men constitute the primary perpetrators of violence against women, including within higher education institutions. An integrative review on the prevalence and associated factors of gender-based violence in universities identified that the majority of violence experienced by female students is perpetrated by men, particularly intimate partners, boyfriends, or spouses^(72)^. Furthermore, a US-based study revealed that 20% to 26% of male university students reported a propensity to perpetrate violence, with rape myth acceptance being positively associated with this likelihood^(73)^.

Moreover, the results point to a predominance of strategies targeting mixed-gender groups, in addition to those tailored exclusively for men or women. Consequently, planning supplementary training components specifically tailored to each group can reveal the potential of each of these strategies. While mixed- gender environments foster collective solidarity and collaborative relationships, activities designed exclusively for men provide opportunities to enhance engagement, address the influence of patriarchy, and promote social accountability. Additionally, activities exclusively for women can offer a more welcoming and intimate space for sharing lived experiences of violence.

This scoping review demonstrates that **bystander training focuses predominantly on sexual violence prevention**. While efforts to prevent on-campus sexual violence span various regions globally, the evidence mapped in this study remain heavily concentrated in the USA. Concurrently, in Latin America, there is a rising institutional concern across universities to develop protocols to prevent this type of violence^(74)^.

Furthermore, a literature review utilizing Google Scholar demonstrated a correlation between core keywords and the publication language of the articles. In articles published in Spanish, the term ‘gender-based violence’ was most frequent, which encompasses different forms of violence, ranging from broad concepts such as ‘violence against women’ to specific manifestations such as sexual violence, physical assault, harassment, and abuse. In studies published in English, ‘sexual violence’ emerged as the predominant focus, being one of the most studied forms of violence within higher education institutions in Anglophone countries^(75)^. This is consistent with the findings of the present scoping review, which highlight a predominance of US-based research on sexual violence prevention strategies.

However, focusing on a single type of violence can be limiting, especially considering the overlap of different types of violence. Sexual violence, for instance, occurs not only within intimate partner relationships, but also across other relational contexts, where the perpetrator may be a stranger, acquaintance, or friend^(70)^. Ultimately, gender-based violence must be understood as a social phenomenon rooted in patriarchal relations, anchored in an unequal power dynamic of men over women that operates in all areas of society^(76)^, including higher education institutions.

This intertwining of gender-based violence with the social subordination of women cannot be understood or addressed in isolation from the broader structural violence inherent to capitalist society. Therefore, it is necessary to coordinate struggles and actions to dismantle the social relations and institutional frameworks that oppress women^(77)^. This indicates the necessity of designing more comprehensive prevention programs that address different types of violence while accounting for the context of social relationships among university students.

The findings regarding the various **types of prevention programs** align with the results of a meta-analysis of college dating violence prevention programs. The strategies analyzed in the study featured active participation (77%) and/or group discussions (55%), a didactic presentation style (39%), group activities (36%) and/or an online component (29%)^(70)^.

Group discussions offer an opportunity for students to connect with peers, fostering a sense of collective responsibility and an understanding of gender-based violence as a social issue in universities. The results of a qualitative study on sexual violence in Guatemala showed that students feel safer when they act together as a group. Collective action signals to potential victims that they are not alone, as well as shows potential aggressors that their behavior will not be tolerated^(78)^. Ultimately, group sessions can encourage dialogue and active student participation. Conditions for developing critical thinking are created through dialogue, which involves the dialectical movement between doing and thinking about doing, in a process that broadens social awareness^(79)^.

Furthermore, this review identified two game-based strategies. A systematic review examining the use of games as an educational tool among nursing students demonstrated that the desire to win encouraged student participation and introduced realism to the activity, promoting greater engagement and enjoyment, thereby enhancing the learning experience^(80)^.

Regarding the **delivery method of the interventions** identified in this review, most were conducted in person. This aligns with the findings of a scoping review analyzing intimate partner violence prevention strategies among graduate students, where face-to-face interventions were utilized in 80% of the included studies^(81)^. The social dimension of in-person activities helps students to be more engaged with the program content and underscores the importance of the topic. Conversely, students often perceive online programming as abstract and impersonal, and that the independence facilitated by online approaches undermines the program’s effectiveness^(68)^.

Regarding the delivery method, the results of a meta-analysis also indicated that virtual methods, such as the use of videos or exclusively online formats, are less effective for the development of bystander skills. They suggest that in-person interaction in a group setting is more effective in achieving this goal^(70)^.

The **presence of facilitators** was identified in 20 of the 41 included strategies, occurring exclusively in in-person formats. This proportion is slightly lower than the 61% reported in a meta-analysis of dating violence prevention programs in university settings^(70)^. A meta-analysis on the effectiveness of bystander programs that address sexual violence on college campuses showed that the difference between the effects of facilitator-led programs and self-directed programs, such as videos or poster campaigns, was not significant. Both delivery formats had positive effects on bystander attitudes, beliefs, and behaviors. Additionally, the outcomes of facilitator-led formats did not vary based on whether the program was delivered by student peer facilitators, university staff, or other adults^(67)^.

In this review, **the duration and number of sessions** varied substantially across the strategies, precluding a direct data synthesis. However, the findings reveal the predominance of single- session programs, with durations ranging from several minutes to a few hours for most formats, excluding social marketing campaigns, which involved longer exposure times. This pattern aligns with the meta-analysis on college dating violence prevention programs, where 77% of the strategies were delivered in a single session, and the interventions lasted from 18 minutes to 12 hours, with the majority lasting less than two hours^(70)^.

Another review indicated that in-person interventions require longer completion times for participants than online formats. This review also pointed out that many studies did not report essential information about the intervention’s delivery, which made it difficult to critically assess patterns regarding delivery method, duration, and frequency across the studies^(81)^. Therefore, it is important to consider evidence demonstrating that program duration is positively associated with shifts in bystander attitudes and beliefs, meaning that longer interventions yield greater attitudinal effects. However, this direct association did not extend to actual bystander behavior^(67)^.

Although this scoping review does not aim to evaluate the interventions’ efficacy, the findings invite further reflection on program duration and continuity. In this regard, universities can benefit from implementing more extensive programs or sustained preventive actions, including multicomponent approaches. Furthermore, the integration of bystander training into academic curricula^(82)^, combined with evidence-based continuous education for the community^(83)^, represents a recommended strategy for building a safe and equitable university environment.

Therefore, it is important to analyze the **topics covered in the training sessions**. This review found that most interventions focused on bystander attitudes, skills, and interventions. Other common themes included rape myths and gender stereotypes, definitions, concepts, and epidemiology of violence, consent and healthy communication. Content related to awareness, personal values and ethics, available resources, and risks, barriers, and obstacles to intervention were less frequent.

These findings align with existing literature. A meta-analysis showed that the most common content addressed in the strategies focused on skill-building (81%) and bystander training (74%). Other common components comprised discussions of types of violent behaviors (42%), the prevalence of dating and/or sexual violence (39%), the definition of consent (39%), available campus resources (36%), and consequences of dating and/or sexual violence (32%)^(70)^.

Furthermore, a review investigating bystander intervention programs for sexual violence prevention in universities found that the most common themes were bystander behavior, likelihood to intervene, efficacy, confidence and willingness to act, and changes in attitudes, beliefs, and norms^(84)^. The predominance of content focusing on bystander attitudes, skills, and interventions (along with rape myths and gender stereotypes) aligns with the core objectives of bystander-centered strategies, which aim to empower students to play an active role in reducing gender-based violence.

The second most frequent content category involved definitions, concepts, and the epidemiology of violence. Evidence suggests that programs including local prevalence data had greater effects on bystander efficacy and intentions to prevent dating violence. The study also indicated that learning the high rates of violence on campus can raise students’ awareness about the severity of the problem and the need to reduce its prevalence, encouraging participants to intervene^(70)^. This demonstrates that gender-based violence prevention programs can benefit from incorporating local epidemiological data. Consequently, using culturally contextualized definitions helps participants recognize violent situations and behaviors.

Information on available resources and services for students experiencing violence was addressed in only five studies in this review. One of these studies, conducted with undergraduate students across three universities, showed that nearly half of the participants were unaware of the resources available on their campus^(85)^. This indicates a limitation in current prevention strategies, as a lack of information about institutional support services can restrict bystander responses to situations of violence.

Conversely, evidence indicates that students’ knowledge of campus policies and procedures regarding sexual assault correlates significantly with intentions to engage in pro-social bystander behaviors and/or attitudes^(86)^. Universities should therefore formalize institutional policies to prevent gender-based violence, ensuring that the academic community has clear access to information on safety procedures and resources to protect their rights^(82)^.

Content regarding risks, barriers, and obstacles to intervention was identified in only four studies. However, research on domestic and sexual violence found adverse consequences for bystanders who intervened, including reports of harassment, threats, and physical assault^(87)^.

Detailed statistics on the incidence of gender-based violence and the demographics of perpetrators and victims help tailor prevention programs to the specific needs of the university community. Additionally, continuous data collection and analysis are essential to evaluate the effectiveness of these interventions, track specific types of violence, and make necessary adjustments. This process involves analyzing the social and economic structures that sustain violence, as well as promoting interventions aimed at changing these dynamics and creating a safer and more equitable academic environment for all.

One limitation of this study was the lack of complete data regarding the characteristics of the strategies. For instance, the duration of the strategy could not be identified in one article, and the number of sessions was missing in others. The specific objectives of each training program were not systematized.

## CONCLUSION

Bystander training programs aimed at preventing gender-based violence among female university students are primarily concentrated in North American contexts, focusing predominantly on sexual violence. Intervention formats vary widely, including group sessions, marketing campaigns, online courses, workshops, and games, with an emphasis on in-person activities and mixed-gender groups. Most strategies are one-off initiatives and lack integration into institutional policies. Training content frequently covers definitions and concepts of violence, gender myths and stereotypes, consent, and bystander attitudes. Institutional resources and barriers to intervention are rarely addressed in the content of the strategies, which represents a gap that may limit the reach of gender-based violence prevention efforts.

This study highlights the need for comprehensive, continuous interventions that address different forms of gender-based violence. Future initiatives should broaden the scope of their content to include risks, barriers, and institutional resources, while being integrated into permanent prevention policies in universities. These measures contribute to building safer, more equitable academic environments committed to preventing gender-based violence.

## Data Availability

The search strategies used for each information source are available at the following repository: (https://osf.io/x4pnf/files/hkvc6).

## References

[1] Bows H, Fileborn B (2020). Space, place and GVB. J Gend Based Violence.

[2] Steele B, Esposti MD, Mandeville P, Humphreys DK (2023). Sexual violence among higher education students in the United Kingdom: results from the Oxford understanding relationships, sex, power, abuse and consent experiences study. J Interpers Violence.

[3] Reynolds M, Anyadike-Danes N, Lagdon S, Aventin Á, Armour C (2023). Prevalence of unwanted sexual experiences and their associations on university students in the United States, United Kingdom, and Ireland: a systematic review. J Sex Aggress.

[4] Workye H, Mekonnen Z, Wedaje W, Sitot A (2023). Prevalence and predictors of gender-based violence among Wolkite University female students, southwest Ethiopia, 2021: cross-sectional study. Front Reprod Health.

[5] Jones C, Farrelly N, Barter C (2025). UK prevalence of university student and staff experiences of sexual violence and domestic violence and abuse: a systematic review from 2002 to 2022. European Journal of Higher Education.

[6] Wood L, Voth Schrag R, Busch-Armendariz N (2020). Mental health and academic impacts of intimate partner violence among IHE-attending women. J Am Coll Health.

[7] Molstad TD, Weinhardt JM, Jones R (2021). Sexual assault as a contributor to academic outcomes in university: a systematic review. Trauma Violence Abuse.

[8] Mendes K, Horeck T, Ringrose J (2022). Sexual violence in contemporary educational contexts. Gend Educ.

[9] Dufour GK (2024). The insidiousness of institutional betrayal: an ecological systematic review of campus sexual violence response literature. Trauma Violence Abuse.

[10] Bush HM, Bell SC, Coker AL (2019). Measurement of bystander actions in violence intervention evaluation: opportunities and Challenges. Curr Epidemiol Rep.

[11] Banyard VL, Moynihan MM, Crossman MT (2009). Reducing sexual violence on campus: the role of student leaders as empowered bystanders. J Coll Student Dev.

[12] Banyard VL (2015). Toward the next generation of bystander prevention of sexual and relationship violence: Action coils to engage communities.

[13] Carlyle KE, Conley AH, Guidry JPD (2022). Development and evaluation of the red flag campaign for the primary prevention of sexual and dating violence on college campuses. J Am Coll Health.

[14] Feldwisch RP, Whiston SC, Arackal IJ (2020). Safe sisters: a sorority-based bystander intervention program to prevent sexual assault. J Coll Couns.

[15] Heard E, Evans C, Buckley L, Hatchman K, Masser B (2024). Evaluating an online module for sexual violence prevention in a tertiary educational setting: an exploratory study. Health Promot J Austr.

[16] Johnson N, Phillips M (2018). Rayyan for systematic reviews. J Electron Resour Librariansh.

[17] Tricco AC, Lillie E, Zarin W, O’Brien KK, Colquhoun H, Levac D (2018). PRISMA Extension for Scoping Reviews (PRISMA-ScR): checklist and Explanation. Ann Intern Med.

[18] Peters MDJ, Godfrey C, McInerney P, Munn Z, Tricco AC, Khalil H, Aromataris E, Lockwood C, Porritt K, Pilla B, Jordan Z (2024). JBI manual for evidence synthesis.

[19] Pollock D, Peters MDJ, Khalil H, McInerney P, Alexander L, Tricco AC (2023). Recommendations for the extraction, analysis, and presentation of results in scoping reviews. JBI Evid Synth.

[20] Elo S, Kyngäs H (2008). The qualitative content analysis process. J Adv Nurs.

[21] Bannon RS, Foubert JD (2017). The bystander approach to sexual assault risk reduction: effects on risk recognition, perceived self-efficacy, and protective behavior. Violence Vict.

[22] Banyard V, Potter SJ, Cares AC, Williams LM, Moynihan MM, Stapleton JG (2018). Multiple sexual violence prevention tools: doses and boosters. J Aggress Conflict Peace Res.

[23] Bonar EE, Rider-Milkovich HM, Huhman AK, McAndrew L, Goldstick JE, Cunningham RM (2019). Description and initial evaluation of a values-based campus sexual assault prevention programme for first-year college students. Sex Educ.

[24] Borsky AE, McDonnell K, Turner MM, Rimal R (2016). Raising a red flag on dating violence: evaluation of a low-resource, college-based bystander behavior intervention program. J Interpers Violence.

[25] Cadaret MC, Johnson NL, Devencenzi ML, Morgan EM (2021). A quasiexperimental study of the bystander plus program for changing rape culture beliefs. J Interpers Violence.

[26] Coker AL, Bush HM, Fisher BS, Swan SC, Williams CM, Clear ER (2016). Multi-college bystander intervention evaluation for violence prevention. Am J Prev Med.

[27] Donais L, Simonsen B, Simonsen N (2018). Gender-based violence prevention workshops: an experimental evaluation of efficacy. Int J Public Adm.

[28] Doran F, Orrock P (2021). Educating university allied health students about gender-based violence: report of a pilot study. FoHPE.

[29] Exner-Cortens D, Cummings N (2021). Bystander-based sexual violence prevention with college athletes: a pilot randomized trial. J Interpers Violence.

[30] Hahn CK, Morris JM, Jacobs GA (2017). Predictors of bystander behaviors and sexual assertiveness among college women attending a sexual assault prevention program. J Community Psychol.

[31] Hetzel-Riggin MD (2018). An exploratory evaluation of bystander intervention training for resident assistants. Violence Gend.

[32] Hust SJT, Adams PM, Willoughby JF, Ren C, Lei M, Ran W (2017). The entertainment-education strategy in sexual assault prevention: a comparison of theoretical foundations and a test of effectiveness in a college campus setting. J Health Commun.

[33] Inman EM, Chaudoir SR, Galvinhill PR, Sheehy AM (2018). The effectiveness of the bringing in the BystanderTM program among first-year students at a religiously-affiliated liberal arts college. J Soc Polit Psych.

[34] Johnson KM, Lederer AM, Liddell JL, Sheffield S, McCraw A (2021). Teaching to impact sexual violence? The evaluation of a curricular intervention for first-year college students. Am J Health Promot.

[35] Jouriles EN, McDonald R, Rosenfield D, Levy N, Sargent K, Caiozzo C (2016). TakeCARE, a Video bystander program to help prevent sexual violence on college campuses: results of two randomized, controlled trials. Psychol Violence.

[36] Jouriles EN, Kleinsasser A, Rosenfield D, McDonald R (2016). Measuring bystander behavior to prevent sexual violence moving beyond self reports. Psychol Violence.

[37] Jouriles EN, Sargent KS, Salis KL, Caiozzo C, Rosenfield D, Cascardi M (2020). TakeCARE, a video to promote bystander behavior on college campuses: replication and extension. J Interpers Violence.

[38] McCall D, Elhindi J, Krogh C, Chojenta P, Lampis M, Phelan L (2020). Creating cultural change: sex, safety and respect workshops as one response to sexual assault and harassment on campus. JANZSSA.

[39] McKendrick W (2020). Interpersonal violence among african american young adult women and the violence interruption process as a bystander intervention. J Afr Am Stud.

[40] Mennicke A, Kennedy SC, Gromer J, Klem-O’Connor M (2021). Evaluation of a social norms sexual violence prevention marketing campaign targeted toward college men: attitudes, beliefs, and behaviors over 5 years. J Interpers Violence.

[41] Nieder C, Bosch JF, Nockemann AP, Kärtner J (2022). Evaluation of RISE: a sexual violence prevention program for female college students in India. J Interpers Violence.

[42] O’Brien KM, Sauber EW, Kearney MS, Venaglia RB, Lemay EP (2021). Evaluating the effectiveness of an online intervention to educate college students about dating violence and bystander responses. J Interpers Violence.

[43] Peterson K, Sharps P, Banyard V, Powers RA, Kaukinen C, Gross D (2018). An evaluation of two dating violence prevention programs on a college campus. J Interpers Violence.

[44] Potter SJ, Flanagan M, Seidman M, Hodges H, Stapleton JG (2019). Developing and piloting videogames to increase college and university students’ awareness and efficacy of the bystander role in incidents of sexual violence. Games Health J.

[45] Potter SJ, Demers JM, Flanagan M, Seidman M, Moschella EA (2021). Can video games help prevent violence? An evaluation of games promoting bystander intervention to combat sexual violence on college campuses. Psychol Violence.

[46] Rothman EF, Paruk J, Banyard V (2018). The escalation dating abuse workshop for college students: results of an efficacy RCT. J Am Coll Health.

[47] Salazar LF (2018). Vivolo-Kantor A, Schipani-McLaughlin AM. Theoretical mediators of realconsent: a web-based sexual violence prevention and bystander education program. Health Educ Behav.

[48] Stojanov Z, Treharne GJ, Graham K, Liebergreen N, Shaw R, Hayward M (2021). Pro-social bystander sexual violence prevention workshops for first year university students: perspectives of students and staff of residential colleges. Kotuitui.

[49] Sundstrom B, Ferrara M, DeMaria AL, Gabel C, Booth K, Cabot J (2018). It’s your place: development and evaluation of an evidence-based bystander intervention campaign. Health Commun.

[50] Thomas KA, Sorenson SB, Joshi M (2016). “Consent is Good, Joyous, Sexy”: A banner campaign to market consent to college students. J Am Coll Health.

[51] Wong JY, Tang NR, Yau JH, Choi AW, Fong DY (2019). Dating CAFE ambassador programme: chinese college students to help peers in dating violence. Health Educ Behav.

[52] Wong YJ, McDermott RC, Zounlome NOO, Klann EM, Peterson ZD (2022). Self-persuasion: an experimental evaluation of a sexual aggression preventive intervention for U.S. college men. J Interpers Violence.

[53] Yount KM, Bergenfeld I, Anderson KM, Trang QT, Sales JM, Cheong YF (2022). Theoretical mediators of GlobalConsent: an adapted web-based sexual violence prevention program for university men in Vietnam. Soc Sci Med.

[54] Yount KM, Cheong YF, Bergenfeld I, Trang QT, Sales JM, Li Y (2023). Impacts of globalconsent, a web-based social norms edutainment program, on sexually violent behavior and bystander behavior among university men in Vietnam: randomized controlled trial. JMIR Public Health Surveill.

[55] Zapp D, Buelow R, Soutiea L, Berkowitz A, DeJong W (2021). Exploring the potential campus-level impact of online universal sexual assault prevention education. J Interpers Violence.

[56] Barter C, Bracewell K, Farrelly N, Clelland AK, Chantler K (2025). Prevention of sexual violence and domestic abuse through a university bystander intervention programme: learning from a UK feasibility study. J Gend Based Violence.

[57] Bloom TL, Perrin N, Brown ML, Campbell J, Clough A, Grace KT (2023). Concerned friends of intimate partner violence survivors: results from the myPlan randomized controlled trial on college campuses. BMC Public Health.

[58] Cadaret MC, Ritter M, Kohnen S, Bergman Z, Folio F, Albrecht J (2024). Evaluation of the define it! program for raising critically conscious bystander behaviors. Violence Against Women.

[59] Carter-Snell CJ, Warthe GD (2023). “Stepping Up”: a decade of relationship violence prevention. Soc Sci.

[60] Chiang ES, Logan RA (2024). Stop the silence: a selfpaced program on bystander intervention for domestic violence for college students. Trends Psychol.

[61] Jaffe N, Jones MC, Angelone DJ (2023). Development, feasibility, and acceptability of sport: a dating violence and sexual risk prevention intervention for college student-athletes. Pilot Feasibility Stud.

[62] Nieder C, Thomae K, Kärtner J (2024). Online sexual violence prevention on a female college campus in India: evaluation of the RISE-ON program. Int J Clin Health Psychol.

[63] Orchowski LM, Zinzow H, Thompson M, Wood S (2023). Open pilot trial of an interactive digital application for campus sexual violence prevention. J Community Psychol.

[64] Steele B, Martin M, Sciarra A, Melendez-Torres GJ, Degli Esposti M, Humphreys DK (2023). The prevalence of sexual assault among higher education students: a systematic review with meta-analyses. Trauma Violence Abuse.

[65] Villalonga-Aragón M, Martí-Vilar M, Merino-Soto C, Tantalean-Terrones L (2023). A scoping review of educational interventions to increase prosociality against gender-based violence in university bystanders. Soc Sci (Basel).

[66] Office for Civil Rights (2015). Title IX resource guide.

[67] Jouriles EN, Krauss A, Vu NL, Banyard VL, McDonald R (2018). Bystander programs addressing sexual violence on college campuses: A systematic review and meta-analysis of program outcomes and delivery methods. J Am Coll Health.

[68] Philyaw-Kotov ML, Walton MA, Brenneman B, Gleckman-Krut M, Davis AK, Bonar EE (2021). What undergraduates want in campus sexual assault prevention programming: findings from a formative research study. J Am Coll Health.

[69] Graham LM, Embry V, Young BR, Macy RJ, Moracco KE, Reyes HLM (2021). Evaluations of prevention programs for sexual, dating, and intimate partner violence for boys and men: a systematic review. Trauma Violence Abuse.

[70] Wong JS, Bouchard J, Lee C (2021). The effectiveness of college dating violence prevention programs: a meta-analysis. Trauma Violence Abuse.

[71] Kikooma J, Kyomuhendo GB, Muhanguzi FK, Babalanda S (2023). Engaging men in gender transformative work in institutions of higher learning: A case of the men’s hub at Makerere University. Front Sociol.

[72] Tassinari TT, Honnef F, Arboit J, Langendorf TF, Paula CC, Padoin SMM (2022). Violência de gênero em mulheres estudantes universitárias: evidências sobre a prevalência e sobre os fatores associados. Acta Colomb Psicol.

[73] Palmer JE, McMahon S, Fissel E (2021). Correlates of incoming male college students’ proclivity to perpetrate sexual assault. Violence Against Women.

[74] Linhares Y, Fontana J, Laurenti C (2021). Protocolos de prevenção e enfrentamento da violência sexual no contexto universitário: uma análise do cenário latino-americano. Saude Soc.

[75] Visbal LA (2023). Artiles. Las relaciones y la violencia basada en género: posibilidades o límites en el campus universitario. Sapientia Technological.

[76] Saffioti H (2015). Gênero, patriarcado, violência.

[77] Aruzza C, Bhattacharya T, Fraser N (2019). Feminismo para os 99%: um manifesto.

[78] Lyons M, Gómez LDR, Chopen N, Dávila N (2024). Student experiences of sexual violence as targets and bystanders - A qualitative investigation in a public university in Guatemala. Sex Cult.

[79] Freire P (2019). Pedagogia da autonomia: saberes necessários à prática educativa.

[80] Xu Y, Lau Y, Cheng LJ, Lau ST (2021). Learning experiences of game-based educational intervention in nursing students: a systematic mixed-studies review. Nurse Educ Today.

[81] An S, Welch-Brewer C, Tadese H (2024). Scoping review of intimate partner violence prevention programs for undergraduate college students. Trauma Violence Abuse.

[82] Paat YF, Mangadu T, Payan SL, Flores SC (2024). Rethinking the roles of the social determinants of health in bystander intervention for partner violence among college students. Societies.

[83] Serradell O, Puigvert L (2025). Overcoming sexual harassment at university: the case of the training intervention in the Universitat Autònoma de Barcelona. Behav Sci.

[84] Pfaff J, Jönsson S, Muhonen T (2024). Bystander intervention programs focusing on sexual violence in academia - A scoping review. SAGE Open.

[85] Bloom BE, Park E, Swendeman D, Oaks L, Sumstine S, Amabile C (2022). Opening the “Black Box”: student-generated solutions to improve sexual violence response and prevention efforts for undergraduates on college campuses. Violence Against Women.

[86] Toews ML, Spencer C, Anders KM, Taylor L (2020). The role of campus environment on bystander intentions and behaviors. J Am Coll Health.

[87] Moschella E, Banyard V (2021). Action and reaction: the impact of consequences of intervening in situations of interpersonal violence. J Interpers Violence.

