# Supplementary material for: Bystander training to prevent gender-based violence against female university students: a scoping review
Source: Rev Esc Enferm USP. 2026 Aug 3;60:e20250231. doi: 10.1590/1980-220X-REEUSP-2025-00231en (PMC13456584; doi:10.1590/1980-220X-REEUSP-2025-00231en)
Supplement: Translation

# Translation: Treinamento de espectadores para prevenir a violência de gênero em mulheres estudantes universitárias: revisão de escopo*

**DOI:** 10.1590/1980-220X-REEUSP-2025-00231pt

**Authors:** Taís Tasqueto Tassinari, Letícia de Mello Padoin, Laís Alves Molina, Cristiane Cardoso de Paula, Stela Maris de Mello Padoin

**Affiliations:** 1 Prefeitura Municipal de Penha, Departamento de Vigilância Sanitária, Penha, SC, Brasil.; 2 Universidade Federal de Santa Catarina, Programa de Pós-graduação em Serviço Social, Florianópolis, SC, Brasil.; 3 Universidade Franciscana, Residência Profissional em Enfermagem Obstétrica, Santa Maria, RS, Brasil.; 4 Universidade Federal de Santa Maria, Programa de Pós-graduação em Enfermagem, Santa Maria, RS, Brasil.

**Keywords:** Prevenção, Universidades, Violência de Gênero, Revisão de Escopo, Mulheres

## Abstract

**Objetivo:**

Mapear as características dos treinamentos de espectadores para prevenir a violência de gênero em mulheres estudantes no contexto universitário.

**Método:**

Revisão de escopo pelo método do JBI. A busca foi realizada em março/2025 no MEDLINE/PubMed, WoS, BVS, EBSCO, EMBASE, Scopus, PsycInfo/APA e SciELO, independentemente do idioma, com o recorte temporal de 2016. A seleção e extração foram duplo-independentes, com análise descritiva da caracterização e abordagem indutiva do conteúdo dos treinamentos.

**Resultados:**

Foram identificados 9 tipos de treinamentos em 46 estudos incluídos que visavam prevenir a violência sexual usando abordagem mista de gênero. O conteúdo dos treinamentos foi categorizado em: definições, conceitos e epidemiologia da violência; mitos do estupro e estereótipos de gênero; consentimento e comunicação saudável; atitudes, comportamentos e intervenções de espectadores; recursos disponíveis e serviços; valores, consciência e ética pessoal; riscos, barreiras e obstáculos à intervenção.

**Conclusão:**

A lacuna reside na concentração temática dos estudos, focados na violência sexual, com limitada exploração de riscos, barreiras à intervenção e recursos institucionais nos conteúdos das estratégias.

## INTRODUÇÃO

A violência de gênero é um problema global observado em contextos sociais e econômicos enraizados em relações de poder desiguais, com estrutura patriarcal, afetando principalmente as mulheres^(1)^. Elas são as mais prejudicadas no contexto universitário e a prevalência de violência sexual entre estudantes pode ser maior que na população geral^(2)^. Um estudo de revisão com estudantes universitários encontrou taxas de prevalência variando de 1,3% a 88%, com ampla variação conforme o tipo de violência, a ferramenta de coleta de dados e a amostra dos estudos^(3)^.

A violência de gênero entre estudantes universitárias expressa-se de diversas maneiras; entre os agressores estão seus parceiros e ex-parceiros, colegas e outros estudantes, professores e funcionários das universidades^(4)^. A violência que elas sofrem está diluída em seu cotidiano, ocorrendo em ambientes domésticos (tais como suas casas e dos agressores), locais da universidade (tais como sala de aula, espaços de convívio comum no *campus*) e festas^(5)^.

A violência contra universitárias impacta gravemente a saúde física e mental, bem como o desempenho acadêmico. Violência por parceiros íntimos pode desencadear quadros de depressão e estresse pós-traumático, cuja gravidade está significativamente correlacionada à intensidade da violência^(6)^. Além dos sintomas de ansiedade e comportamento suicida devido à violência sofrida, os resultados de uma revisão sistemática mostraram que a violência sexual foi associada a médias de notas mais baixas, absenteísmo e abandono escolar^(7)^.

A normalização e banalização da violência em espaços educacionais é um agravante às mulheres que enfrentam essa situação. Frequentemente, as experiências de violência de gênero são silenciadas e os discursos das vítimas são invalidados para preservar a imagem institucional^(8)^. O processo de “traição institucional” está relacionado às estruturas sociais de poder e opressão, contribuindo para perpetuar a violência. Isso se manifesta em vários níveis institucionais na forma de políticas que dificultam relatos de violência, burocratizam processos, recusam afastar ou remover agressores de alto *status* e na falta de engajamento para promover mudanças sistêmicas e duradouras nas universidades^(9)^.

Para melhor compreender as possibilidades de enfrentar a violência de gênero no espaço universitário, é relevante mapear estratégias preventivas que promovem envolvimento social para responsabilização e transformação cultural dos papéis de gênero. Neste sentido, a literatura aponta para intervenções com treinamento de espectadores; elas partem do pressuposto de que líderes podem fomentar mudanças sociais em comunidades para responder a um problema de saúde pública, p.ex., violência de gênero. O termo espectadores refere-se a pessoas que presenciaram o antes, durante ou depois de um evento violento. Assim, o treinamento de espectadores visa treinar pessoas para que elas possam intervir em atos violentos ou normas sociais que perpetuam violência, buscando prevenir sua ocorrência^(10)^.

Alguns fatores podem promover comportamentos adequados em espectadores para que suas ações sejam eficazes, resultando em prevenção da violência. Alguns exemplos são os seguintes: conteúdos e mensagens que ajudam espectadores a identificar situações de violência; desenvolvimento de habilidades e prática de espectadores para aumentar seu senso de efetividade usando modelos que mostram intervenções que eles podem reproduzir; e abordagem de intervenção que respeita e considera a própria segurança do espectador para que os benefícios de intervir superem as barreiras^(11)^.

Assim, o treinamento de espectadores fortalece habilidades para reduzir o risco de violência, reconhecendo situações que podem se tornar violentas, intervindo com segurança e efetividade e manifestando-se contra atitudes que estimulam ou toleram comportamentos violentos. O treinamento visa reduzir a violência alterando normas sociais e promovendo mudanças nas atitudes e comportamentos dos espectadores, incluindo suas crenças e intenções de como agir. Assim, a passividade frente à violência pode ser desestimulada como uma estratégia para remodelar e estimular a responsabilidade social, prevenindo a violência de gênero^(12)^.

A literatura mostra que há vários estudos sobre treinamento de espectadores, indicando distintos formatos de intervenção^(13,14,15)^. Porém, o esforço para compreender e sistematizar essas estratégias de prevenção ainda é fragmentado. Os dados são dispersos quanto aos métodos e suas características, tais como duração, conteúdo, formato e contexto de aplicação das intervenções. Após identificar essa lacuna, veio a necessidade de mapear as evidências sobre treinamentos para prevenir a violência de gênero, sinalizando assim a necessidade de uma revisão.

Além disso, foi realizada busca preliminar de estudos sobre o tema e não foram identificadas revisões de escopo ou protocolos em andamento no *International Prospective Register of Systematic Reviews* (PROSPERO)*, Open Science Framework* (OSF)*, Medical Literature Analysis and Retrieval System Online* (MEDLINE), *the Cochrane Database of Systematic Reviews*, e JBI *Evidence Synthesis*. Essa situação confirma a lacuna do conhecimento no tema e a necessidade de desenvolver uma revisão de escopo. Portanto, o objetivo desta revisão foi mapear as características dos treinamentos de espectadores para prevenir a violência de gênero em mulheres estudantes no contexto universitário.

## MÉTODO

### Desenho do Estudo

Esta revisão de escopo foi conduzida segundo o método do JBI^(16)^ e relatada conforme o *Preferred Reporting Items for Systematic reviews and Meta-Analyses extension or Scoping Reviews* (PRISMA-ScR)^(17)^. O protocolo foi registrado na *Open Science Framework* (https://doi.org/10.17605/OSF.IO/4NK9U).

### Identificação da Pergunta de Revisão

A pergunta de revisão (“Quais as características dos treinamentos de espectadores para prevenir a violência de gênero em mulheres estudantes no contexto universitário?”) foi estruturada a partir do acrônimo PCC; ele indica população (**P**: estudantes universitários), conceito (**C**: prevenção da violência de gênero entre mulheres estudantes universitárias, usando treinamento de espectadores) e contexto (**C**: *campi* das universidades).

A partir dessa pergunta, foram elaboradas as questões secundárias: Quais são os tipos de intervenção retratados na literatura? Qual a forma de entrega das intervenções? As intervenções têm ou não a presença de um mediador? Qual é a duração das intervenções? Quantas sessões ou etapas têm as intervenções? Qual o conteúdo das intervenções?

### Critérios de Elegibilidade

Participantes: foram elegíveis os estudos realizados com estudantes universitários (de ambos sexos e gêneros, sem restrição de idade) matriculados em cursos de graduação ou pós-graduação. A revisão contemplou amostras (tanto gerais como específicas) de estudantes universitários, como estudos só com mulheres, graduandos, pós-graduandos ou membros de irmandades e/ou fraternidades.

Conceito: foram considerados elegíveis estudos que descreveram treinamento de espectadores voltados para prevenir violência de gênero contra mulheres universitárias (cisgênero e transgênero), abrangendo violência de parceiro íntimo, física, sexual, psicológica, moral ou patrimonial. As intervenções poderiam ser apresentadas em vários formatos (programas educativos, vídeos, treinamentos *online*, campanhas, aulas ou palestras), sem limitação sobre duração ou número de sessões, podendo ou não incluir facilitadores. Foram excluídos estudos focando estratégias profissionais de prevenção de violência, autodefesa, e aqueles combinando prevenção de violência com prevenção de Infecções Sexualmente Transmissíveis (ISTs) ou uso abusivo de álcool, devido à complexidade para sintetizar esses temas.

Contexto: foram elegíveis os estudos realizados no *campus* das universidades públicas ou privadas em qualquer país, e via plataforma digital. Foram excluídos estudos sobre intervenção realizada fora das instituições educacionais, mesmo que apoiados por elas.

Tipo de estudo: foram elegíveis os desenhos de estudos experimentais e quase-experimentais, incluindo estudos controlados (antes e depois) e estudos de séries temporais interrompidos. Esta revisão também considerou estudos observacionais, qualitativos e mistos. Após uma busca exploratória, identificou-se que a produção de literatura cinzenta tinha limitações quanto à apresentação dos treinamentos e descrição metodológica, comprometendo a consistência na clareza, seleção e completude para extrair dados. Assim, por exemplo, foram excluídos editoriais, artigos de opinião ou reflexão, boletins epidemiológicos, legislações estatais e federais, guias, cartilhas e notas técnicas.

Em relação ao ano de publicação dos estudos, considerou-se um marco temporal, o Dia Internacional das Mulheres e Meninas nas Ciências, em 11 de fevereiro, aprovado pela Assembleia das Nações Unidas em 22 de dezembro de 2015. Embora o marco seja simbólico e político, sua adoção como critério metodológico justifica-se por sinalizar a necessidade de vigência do compromisso global de mudança de paradigma para garantir a igualdade de gênero em ambientes de pesquisa. Portanto, foram elegíveis para esta revisão os estudos publicados a partir de 2016, alinhando o novo paradigma de proteção das estudantes no espaço acadêmico com o levantamento bibliográfico de uma ação prática, como o treinamento de espectadores, com período de mobilização institucional para enfrentamento de violências em contexto de ensino e pesquisa.

### Estratégia de Busca e Fontes de Informação

Seguindo as recomendações do JBI, a estratégia de busca ocorreu em três etapas. A etapa 1 consistiu em uma busca limitada à base de dados *Medical Literature Analysis and Retrieval System Online* (MEDLINE/PubMed), onde foram analisadas as palavras-chave em títulos e resumos dos artigos identificados.

Após este mapeamento de termos, foram desenvolvidas as estratégias de busca nas outras fontes de informação (etapa 2).

As fontes de informação consultadas para recuperar estudos incluem 8 bases de dados e bibliotecas virtuais: *Medical Literature Analysis and Retrieval System Online* (MEDLINE/PubMed); *Web of Science Core Collection* (WoS); Biblioteca Virtual em Saúde (BVS); EBSCO; EMBASE; *SciVerse Scopus* (Scopus); *PsycInfo* (APA) e *Scientific Electronic Library Online* (SciELO). Foram incluídos estudos publicados em qualquer idioma. Todas as buscas foram realizadas em março de 2023 e atualizadas em fevereiro-março de 2025. A etapa 3 consistiu em uma pesquisa na lista de referências, mas nenhuma referência foi incluída nesta etapa. Todas as etapas foram realizadas com a colaboração de uma bibliotecária especialista em estudos de revisão na área da saúde.

### Seleção dos Estudos

As referências foram agrupadas e carregadas no *software Endnote* 20/2020 (*Clarivate Analytics*; PA, EUA); após a remoção de duplicatas, elas foram exportadas para o sistema Rayyan (*Qatar Computing Research Institute*, QCRI)^(18)^. Títulos e resumos foram selecionados para avaliação por duas revisoras (modo duplo independente) de acordo com os critérios de elegibilidade. Estudos potencialmente relevantes foram recuperados integralmente e avaliados detalhadamente pelas revisoras (mantendo o processo independente) conforme os critérios de elegibilidade. Os motivos para exclusão de estudos de texto completo foram registrados e relatados. Em cada etapa do processo de seleção, as divergências que surgiram entre as revisoras foram resolvidas discutindo com a terceira revisora, que era *expert* no tema violência de gênero. Os resultados de busca e processo de seleção dos estudos foram relatados integralmente e apresentados em um diagrama de fluxo^(17)^.

### Extração dos Dados e Apresentação dos Resultados

A extração das evidências foi multifásica. Para a fase de caracterização dos estudos foi usada a ferramenta padronizada: país, ano, periódico, população, objetivo e método dos estudos^(16)^. Para a fase de mapeamento do conceito, foram extraídos os dados seguintes: tipo de intervenção (apresentação de pôsteres, vídeo, curso *online*, jogo educativo etc.); conteúdo da intervenção; características das intervenções (duração, presença ou não de mediador, número de sessões e/ou etapas, tipo específico de violência que visa prevenir) e forma de entrega da intervenção (presencial ou *online*). Ressalta-se que, em sete estudos, foram identificadas duas intervenções diferentes e uma mesma intervenção foi evidenciada em estudos distintos, o que resultou no quantitativo de estudos incluídos ser diferente do total de estratégias mapeadas.

### Análise dos Dados

Foi usada análise descritiva para caracterizar os estudos primários e as características dos treinamentos, usando frequência simples. A apresentação desses resultados (incluindo figuras e quadros) foi organizada graficamente, acompanhados de sua descrição. Para analisar o conteúdo dos treinamentos, usou-se a abordagem indutiva de análise de conteúdo qualitativa básica; esta é uma abordagem descritiva de análise envolvendo codificação aberta para alocar características em categorias gerais^(19)^. Esta análise foi realizada em três fases: 1) preparação: familiarização com os dados lendo as fontes de evidência para identificar o conteúdo das estratégias de treinamentos de espectadores, visando prevenir a violência de gênero em mulheres estudantes no contexto universitário; 2) organização: identificação dos tópicos de cada estratégia e codificação dos conteúdos por semelhança em categorias; 3) relato: apresentação descritiva e gráfica dos resultados^(20)^.

A avaliação da qualidade metodológica não foi realizada, considerando a distinção entre revisões sistemáticas e de escopo, em que a primeira delas deve incluir avaliação crítica ou do risco de viés para recomendar os resultados sintetizados na implementação em políticas ou práticas. Em contraste, não é obrigatório que as revisões de escopo avaliem a qualidade das evidências incluídas, mas deem uma visão geral da fronteira do conhecimento para recomendar a descrição das evidências mapeadas a fim de implementar pesquisas sobre as lacunas do conhecimento^(16)^.

## RESULTADOS

As referências selecionadas foram agrupadas e carregadas no *software* gerenciador de referências Endnote 20/2020. As duplicatas foram removidas; em seguida, as referências restantes foram exportadas para a plataforma *online* Rayyan, onde foi realizada a leitura de títulos e resumos. Um total de 127 estudos foram selecionados para leitura integral aplicando os critérios de elegibilidade. Destes, 81 foram excluídos por não atenderem aos critérios de elegibilidade; assim, 46 estudos compuseram o corpo desta revisão (Figura 1).

**Figura 1:** Fluxograma Preferred Reporting Items for Systematic Reviews and Meta-analyses extension for scoping review (PRISMA-ScR – Santa Maria, RS, Brasil, 2025).

O país de realização dos estudos, ano de publicação, objetivo, tipo de estudo, número e gênero dos participantes são mostrados no Quadro 1. Nos estudos primários, não foi identificada uniformidade na forma de apresentação das informações referentes ao gênero dos participantes. Sua extração seguiu a terminologia empregada em cada estudo. No Quadro 1, essas informações foram sintetizadas e representadas por meio de símbolos, utilizados para fim, especificamente, ilustrativo e de organização do quadro.

**Quadro 1:** Caracterização dos estudos incluídos – Santa Maria, RS, Brasil, 2025.

País e ano	Objetivo	Tipo de estudo e participantes
EUA, 2017^(21)^	Investigar a eficácia do *The Women’s Program*, um programa de conscientização sobre violência sexual voltado para mulheres.	Quase experimental. n:103 ♀
EUA, 2018^(22)^	Examinar como dois métodos de prevenção funcionaram separadamente e em conjunto para promover mudança de atitude relacionada à violência sexual entre estudantes universitários.	Quase experimental. n:948 ♀ ♂
EUA, 2019^(23)^	Fornecer a descrição e dados de avaliação de um programa para prevenir violência sexual, administrado a estudantes do primeiro ano durante o primeiro semestre de uma grande universidade pública.	Quase experimental. n:2.305 ♀ ♂
EUA, 2016^(24)^	Examinar se uma intervenção breve e fácil de implementar em toda universidade poderia melhorar comportamentos, intenções, atitudes, eficácia e normas sociais dos espectadores para prevenir a violência no namoro e compreender quais variáveis estavam mais fortemente associadas aos comportamentos dos espectadores.	Quase experimental. n:288 ♀ ♂
EUA, 2021^(25)^	Examinar a eficácia de uma intervenção denominada programação *“bystander plus”*.	Quase experimental. n:81 ♀ ♂
EUA, 2022^(13)^	Descrever o desenvolvimento e a avaliação de um programa de prevenção primária de violência sexual e no namoro (*Red Flag Campaign*) amplamente difundido.	Quase experimental. n:203 ♀ ♂
EUA, 2016^(26)^	Examinar os efeitos do *Green Dot* nas taxas de vitimização e violência entre estudantes no primeiro ano com dados coletados durante 4 anos (2010–2013).	Quase experimental. n:7.111 ♀ ♂
EUA, 2018^(27)^	Avaliar até que ponto os workshops *Violence Against Women Prevention Program* reduzem a aceitação do mito de estupro por estudantes, aumentam seu conhecimento e compreensão sobre a política de consentimento afirmativo da Universidade e melhoram sua confiança para interpretar pistas relacionadas ao consentimento sexual.	Experimental. n:1.957 ♀ ♂
Austrália 2021^(28)^	Aumentar a sensibilização e o conhecimento pré-registrados dos estudantes de saúde sobre o contexto da violência de gênero e levar os participantes a aprender abordagens de espectadores para intervir eficazmente e reduzir a violência.	Quase experimental. Estudo piloto. n:21♀ ♂
EUA, 2021^(29)^	Avaliar o programa *Wingman 101*, para prevenir violência sexual baseado em espectadores, projetado para atletas universitários masculinos e membros de fraternidades.	Quase experimental. n:80 ♂
EUA, 2020^(14)^	Examinar a eficácia do programa *Safe Sisters* para intervenção em espectadores sobre agressão sexual voltado a membros de irmandades universitárias.	Experimental. n:139 ♀
EUA, 2017^(30)^	Identificar preditores de comportamento de espectadores e assertividade sexual entre mulheres estudantes de irmandades. Avaliar as mudanças em aceitar o mito de estupro, a autoeficácia de espectadores e a assertividade sexual duas semanas após o *The Women’s Program* ter sido fornecido aos membros das irmandades e relatar os comportamentos dos espectadores durante duas semanas após a intervenção.	Quase experimental. n:141♀
Austrália 2024^(15)^	Avaliar um módulo educacional *online* sobre prevenção e resposta à violência sexual projetado e implementado em uma universidade australiana.	Misto. n:113♀ ♂O
EUA, 2018^(31)^	Examinar a eficácia de um treinamento para intervenção em espectadores *Green Dot* modificado para assistentes residentes.	Quase experimental. n:24♀ ♂
EUA, 2017^(32)^	Examinar um programa de prevenção focado em vários aspectos da agressão sexual, incluindo conteúdo sobre consentimento sexual, intervenção de espectadores, denúncia de violência sexual e incidentes de agressão, busca de ajuda após um incidente e apoio às vítimas de agressão sexual.	Quase experimental. n:146♀ ♂
EUA, 2018^(33)^	Adaptar, replicar e testar a generalização do programa *Bringing the Bystander* ^TM^ em uma faculdade jesuíta privada de artes liberais em Massachusetts.	Quase experimental. n:164♀ ♂
EUA, 2021^(34)^	Avaliar se um curso semestral para estudantes no primeiro ano influenciou conhecimentos, atitudes e intenções comportamentais sobre gênero, sexualidade e violência sexual.	Quase experimental. n:109♀ ♂
EUA, 2016^(35)^	Relatar dois ensaios clínicos randomizados avaliando o programa TakeCARE para espectadores de vídeo, projetado para prevenir a violência sexual em *campi* universitários.	Experimental. Estudo 1: n:213 ♀ ♂ Estudo 2: n:211 ♀ ♂
EUA, 2016^(36)^	Apresentar a avaliação de um novo procedimento desenvolvido para medir o comportamento de espectadores e prevenir a violência sexual.	Quase experimental. n:91♀ ♂
EUA, 2020^(37)^	Replicar e ampliar descobertas anteriores sobre os efeitos do vídeo *TakeCARE* no comportamento de espectadores, testando dois métodos de administração do vídeo e examinando os moderadores de seus efeitos no comportamento dos espectadores.	Experimental. n:557 ♀ ♂
Austrália 2020^(38)^	Dar uma visão geral do processo e implementação, desafios e sucessos encontrados em uma universidade australiana ao adotar o workshop credenciado Sex, *Safety and Respect* baseado em evidências.	Relato de experiência. n:118♀ ♂
EUA, 2020^(39)^	Descrever uma campanha de intervenção em espectadores instituída em uma universidade historicamente negra no sudeste dos EUA.	Qualitativo. n:406♀ ♂
EUA, 2021^(40)^	Avaliar a eficácia de uma inovadora campanha de *marketing* de normas sociais implementada durante 5 anos, destinada a envolver homens na prevenção da violência sexual em um grande *campus* universitário público no sudeste dos EUA.	Série temporal. n:4.158 ♂
Índia 2022^(41)^	Avaliar a eficácia do programa *RISE* para prevenir a violência sexual de estudantes universitárias na Índia.	Quase experimental. n:245 ♀
EUA, 2021^(42)^	Avaliar a eficácia da intervenção *online STOP Dating Violence*, desenvolvida para educar estudantes universitários sobre a violência no namoro e intervenções de espectadores em *campi* universitários.	Experimental. n:317♀ ♂
EUA, 2018^(43)^	Comparar a eficácia de um programa de educação de espectadores (90 min) para prevenir a violência no namoro usando um programa tradicional de conscientização sobre o tema, incluindo um grupo de controle (sem intervenção) sobre mudança de atitudes, crenças, eficácia percebida, intenções e comportamentos autorrelatados em estudantes universitários.	Quase experimental. n:1.001♀ ♂
EUA, 2019^(44)^	Relatar a criação de dois videogames para ensinar aos estudantes universitários as habilidades de intervenção em espectadores em situações de violência sexual, relacionamento e perseguição.	Quase experimental. n:305 ♀ ♂
EUA, 2021^(45)^	Avaliar a eficácia de dois jogos de prevenção *online* direcionados a estudantes universitários: (1) perguntas e respostas e (2) aventura que ensinam habilidades de intervenção aos espectadores para prevenir violência sexual.	Experimental. n:227 ♀ ♂
EUA, 2018^(46)^	Testar a eficácia do workshop *Escalation* para prevenir abuso no namoro com uma amostra de estudantes de graduação.	Experimental. n:85♀ ♂
EUA, 2018^(47)^	Examinar o processo pelo qual um programa de prevenção de violência sexual baseado na web (*RealConsent*) evita a violência sexual e melhora o comportamento dos espectadores.	Experimental. n:743♂
Nova Zelândia 2021^(48)^	Apresentar os resultados quantitativos e qualitativos de um processo piloto do workshop *Bring in The Bystander*, desenvolvido para enfrentar o problema da violência sexual nos *campi* universitários adotando uma abordagem de valores comunitários.	Misto. QUAN+qual. Estudo piloto. n:167♀ ♂
EUA, 2018^(49)^	Apresentar a implementação e avaliação de uma campanha baseada em teoria para promover a intervenção ativa de espectadores.	Quase experimental. n:1.505♀ ♂
EUA, 2016^(50)^	Avaliar lembrança, reação e compreensão de uma breve campanha em banner no campus promovendo o consentimento nas relações sexuais, e determinar se a exposição da campanha estava associada ao envolvimento subsequente em atividades relacionadas à educação, conscientização e prevenção de violência sexual.	Misto. n:628 ♀ ♂
China 2019^(51)^	Avaliar a viabilidade do *Dating Compassion, Assessment, reFerral, and Education (CAFE) Ambassador Programme* na China.	Quase experimental. n:85 ♀ ♂
EUA, 2022^(52)^	Avaliar uma breve intervenção *online* para reduzir a agressão sexual e melhorar os comportamentos pró-sociais dos espectadores entre estudantes universitários heterossexuais do sexo masculino.	Quase experimental. n:241♂
Vietnã 2022^(53)^	Adaptar o programa *RealConsent* para prevenir violência sexual (baseado em teoria e na web) para universitários no Vietnã e testar o impacto do programa adaptado *GlobalConsent* neste cenário.	Experimental. n:730♂
Vietnã 2023^(54)^	Testar o impacto do programa *GlobalConsent* no comportamento sexualmente violento e no comportamento pró-social de espectadores entre homens universitários no Vietnã.	Experimental. n:793♂
EUA, 2021^(55)^	Examinar o impacto a curto prazo de um curso para prevenir a violência sexual muito usado para estudantes universitários como uma abordagem preventiva a nível populacional.	Quase experimental. n:167.424 ♀ ♂TO
Inglaterra 2025^(56)^	Explorar a viabilidade de implementar o *“The Intervention Initiative”* em um ambiente universitário no norte da Inglaterra.	Misto. n:21 ♀ ♂ (GF e entrevistas)
EUA, 2023^(57)^	Descrever o impacto do *myPlan* (auxílio à decisão de segurança personalizado) em amigos preocupados (estudantes universitários de qualquer gênero com 18-24 anos de idade), autoidentificados como tendo uma amiga que sofreu violência por Parceiro Íntimo nos últimos 6 meses.	Experimental. n:293 ♀ ♂
EUA, 2024^(58)^	Examinar os efeitos de uma intervenção de conscientização sobre o comportamento do espectador entre estudantes universitários que concluíram um programa de treinamento de espectadores.	Misto. Estudo 1: n:13♀ ♂NB Estudo 2: n:38 ♀ ♂NB
Canadá 2023^(59)^	Usar técnicas meta-analíticas para explorar os dados de um programa presencial para prevenir violência no relacionamento (entregue antes da COVID-19).	Quase experimental. Série temporal. n:244 ♀ ♂NB,T
EUA, 2024^(60)^	Examinar o impacto de um programa autodidata *online* focando a intervenção de espectadores na violência doméstica.	Quase experimental. n:293 ♀ ♂
EUA, 2023^(61)^	Descrever o desenvolvimento do manual *SPoRT* (intervenção para prevenção) e sua avaliação inicial em viabilidade e aceitabilidade.	Misto. n:30 ♀ ♂
Índia 2024^(62)^	Investigar os efeitos a curto prazo dos módulos *RISE-ON* em conhecimento e atitudes de participantes.	Quase experimental. n:244♀
EUA, 2023^(63)^	Desenvolver e conduzir uma avaliação preliminar do novo aplicativo digital *Make a Change* para prevenir a violência sexual na faculdade para estudantes de 2º-4º anos.	Misto. n:105♀ ♂

Legenda: ♀ mulheres;

♂ homens;

NB: não binários; T: transexual; O: outros gêneros; GF: grupos focais.

Sobre os países de publicação, os estudos foram predominantemente realizados nos Estados Unidos da América (n = 35), seguidos por Austrália (n = 3), Índia (n = 2), Vietnã (n = 2), Nova Zelândia (n = 1), China (n = 1), Canadá (n = 1) e Inglaterra (n = 1). Dos 46 estudos incluídos, 34 apresentaram estratégias voltadas a grupos mistos em relação a gênero, 7 só para homens e 5 só para mulheres.

Em relação ao **tipo específico de violência**, os estudos visaram prevenir violência sexual (34), violência doméstica em relacionamentos e por parceiro íntimo (14), e violência interpessoal e de gênero contra mulheres (abordadas isoladamente ou em conjunto; 4).

Dentre os 46 estudos incluídos, foram identificadas 41 estratégias de treinamento (em alguns casos, um mesmo estudo apresentou evidências sobre duas intervenções diferentes e, algumas intervenções apareceram mais de uma vez em estudos distintos). Tais **estratégias foram classificadas em nove tipos**: sessões em grupo (12)^(14,22,23,25,26,29,31,33,39,41,43,48,51,56,58,61)^, apresentações interativas e vídeos (5)^(21,24,30,35,37,42,52)^, campanhas de *marketing* (6)^(13,22,24,40,49,50)^, cursos, módulos e ferramentas educacionais (8)^(15,34,47,53,54,55,59,60,62)^, workshops (4)^(27,28,38,46)^, jogos de videogame (2)^(44,45)^, aplicativos (2)^(57,63)^, simulação em realidade virtual (1)^(36)^ e minirrevista (1)^(32)^ (Figura 2).

**Figura 2:** Identificação das estratégias para prevenir violência de gênero, classificadas por tipo – Santa Maria, RS, Brasil, 2025.

Quanto à classificação sobre a **implementação das 41 estratégias,** elas foram presenciais (25)^(14,21,22,23,24,25,26,27,28,29,30,31,32,33,34,38,39,40,41,43,46,48,50,51,56,58,59,61)^, digitais ou *online* (11)^(15,42,44,45,47,53,54,55,57,60,62,63)^ e mistas, combinando ações presenciais e elementos digitais (5)^(13,35,36,37,49,52)^.

A **presença de facilitadores ou mediadores** foi identificada especificamente em estratégias entregues de forma presencial, incluindo estratégias de sessão em grupo (11)^(14,22,23,25,26,29,31,33,39,41,43,48,51,56,58,61)^, *workshops* (4)^(27,28,38,46)^, apresentações interativas e vídeos (2)^(21,24,30)^ e curso, módulo ou ferramenta educacional (2)^(34,59)^. No total, 20 estratégias informaram a presença de facilitadores ou mediadores.

A **duração e o número de sessões ou apresentações** variaram conforme o tipo de estratégia. As sessões em grupo variaram de 1-8 h em sessões únicas^(14,23,31,33,43,56,58)^, e duas^(25,30,48)^, três^(51)^ ou quatro sessões^(61)^. Dois estudos não informaram o número de sessões^(26,41)^. Todos os *workshops* foram sessões únicas, com duração de 40 min até um turno^(27,28,38,46)^. Os vídeos e apresentações interativas foram apresentações únicas que variaram de 24–90 min^(21,24,30,35,37,42,52)^. As campanhas de *marketing* ficaram expostas em períodos variáveis (uma semana a cinco anos), permitindo muitas visualizações^(13,22,24,40,49,50)^. Os cursos, módulos e ferramentas educacionais tiveram duração variável (45 min a um semestre), em apresentação única^(15,55,62)^ ou divididos em módulos^(34,47,53,54,59)^. Em um dos estudos estas informações não foram encontradas^(60)^. Os *videogames* tiveram duração de 45–60 min^(44,45)^. As minirrevistas foram dispostas em cinco volumes (oito páginas cada), entregues via correio, permitindo muitas visualizações^(32)^. A simulação em realidade virtual foi dividida em sete simulações, durando 2–4 min cada^(36)^. Os dois aplicativos móveis tinham duração completa de 30 min^(63)^ e acesso ilimitado por um ano^(57)^.

Em relação ao **conteúdo das estratégias**, foram identificadas sete categorias: 1. Definições, conceitos e epidemiologia da violência (n = 36); 2. Mitos relacionados ao estupro e estereótipos de gênero (n = 25); 3. Consentimento e comunicação saudável (n = 26); 4. Atitudes, comportamentos e intenções de espectadores (n = 41); 5. Recursos disponíveis e serviços (n = 5); 6. Valores, consciência e ética pessoal (n = 10); e 7. Riscos, barreiras e obstáculos à intervenção (n = 4) (Figura 3).

**Figura 3:** Distribuição quantitativa de estudos identificados por categoria de conteúdo – Santa Maria, RS, Brasil, 2025.

## DISCUSSÃO

A **predominância geográfica** dos estudos selecionados foi dos EUA (76% do total). Esse dado concorda com outras revisões sistemáticas sobre prevalência de agressão sexual e intervenções educacionais sobre violência de gênero, pois ambas continham ~73% de seus estudos primários provenientes dos EUA^(64,65)^. Isso pode ser compreendido como o resultado de políticas federais, tais como *Title IX*, que obriga as universidades apoiadas com recurso federal a desenvolver estratégias de prevenção à violência, além de proibir a discriminação sexual em programas e atividades educacionais^(66)^. Dada a quantidade de estudos, pode-se inferir que a existência da legislação também estimula a produção de dados sobre prevalência de violência nas universidades, bem como estratégias de prevenção.

Em relação aos participantes nos estudos, houve **predominância de estratégias voltadas a grupos mistos em relação a gênero**. Neste sentido, uma revisão sistemática com meta-análise avaliou a eficácia dos programas de espectadores que abordam violência sexual nos *campi* universitários e não encontrou diferença entre os efeitos quando destinados a grupo misto ou do mesmo sexo. Os efeitos foram avaliados para dois desfechos: atitudes e/ou crenças e comportamentos de espectadores^(67)^.

Estratégias voltadas a grupos mistos também se alinham aos desejos de estudantes universitários, conforme os resultados de um estudo sobre suas preferências em relação às estratégias de prevenção de violência no *campus*. Os estudantes percebem os programas direcionados aos grupos mistos como mais inclusivos em relação a gênero e diversidade sexual, além de dissipar estereótipos sobre os papéis que homens e mulheres desempenham em um incidente de agressão sexual^(68)^.

No presente estudo de revisão, seis artigos apresentam estratégias de prevenção destinadas só a grupos do sexo masculino. Evidências provenientes de uma revisão sistemática indica que programas de prevenção direcionados a homens e meninos apresentam resultados promissores na modificação de atitudes, crenças e normas sociais relacionadas à violência^(69)^. Uma hipótese levantada na literatura é que os homens se mostram mais receptivos e confortáveis ao conteúdo dos treinamentos de espectadores e sentem-se menos julgados pelos colegas quando estão em grupos com participantes do mesmo sexo^(70)^. Convém destacar a construção social masculina nas diversas instituições sociais, que resulta em dinâmicas de poder dos homens sobre as mulheres por influência do patriarcado, sendo necessário abandonar os privilégios que lhe são atribuídos com base no gênero. Por isso, os homens podem ter a percepção de que têm algo a perder com a reestruturação social dos sistemas de poder^(71)^.

É importante planejar estratégias para aumentar a participação masculina em programas para prevenir a violência de gênero nas universidades, pois os homens são os principais perpetradores de violência contra mulheres, inclusive em instituições de ensino. Um estudo de revisão integrativa sobre prevalência e fatores associados à violência de gênero na universidade identificou que a maior parte das violências sofridas pelas mulheres é perpetrada por homens, destacando parceiros íntimos, namorados ou companheiros^(72)^. Além disso, um estudo realizado com universitários nos EUA identificou que 20–26% deles relataram alguma propensão para perpetrar violência, e a aceitação de certos mitos de estupro foi positivamente associada à propensão^(73)^.

Ainda, foi identificada nos resultados a predominância de estratégias voltadas a grupos mistos de gênero, além daquelas voltadas a grupos apenas de homens ou apenas de mulheres. Então, planejar atividades e treinamentos complementares formulados especificamente para cada grupo poderá revelar a potencialidade de cada uma dessas estratégias. Infere-se que quando o grupo é misto pode-se propiciar o cultivo de relações coletivas e colaborativas. Ainda, planejar atividades exclusivas para homens pode ser uma forma de atraí-los e a oportunidade de abordar a influência do patriarcado, buscando a responsabilização social pelo problema. Adicionalmente, atividades exclusivas para mulheres podem ser mais acolhedoras e intimistas para trocar experiências sobre vivências de violência.

Nesta revisão de escopo, **o treinamento de espectadores é destinado principalmente à prevenção da violência sexual**; esforços para prevenir a violência sexual na universidade têm sido adotados em várias partes do mundo, embora estudos recuperados tenham sido realizados principalmente nos EUA. Na América Latina, há uma crescente preocupação nas universidades quanto ao desenvolvimento de protocolos para prevenir este tipo de violência^(74)^.

Além disso, uma revisão após busca no Google Acadêmico relacionou as principais palavras-chave ao idioma de publicação dos artigos. Nos artigos publicados em espanhol, a categoria “violência de gênero” mostrou a maior frequência. Nessa categoria, podem ser incluídas diferentes formas de violência, desde categorias genéricas, tais como “violência contra a mulher”, até outras mais específicas, tais como violência sexual, física, assédio, abuso etc. Nos estudos publicados em inglês, destaca-se a predominância da violência sexual, sendo uma das formas de violência mais estudadas nas instituições de ensino superior em países anglófonos^(75)^. Isso é compatível com os dados da presente revisão em relação à predominância de artigos realizados nos EUA sobre estratégias para prevenir a violência sexual.

Contudo, a abordagem de um único tipo de violência pode ser uma limitação, considerando especialmente a sobreposição dos tipos de violência. A violência sexual pode ocorrer dentro de um relacionamento amoroso bem como em outros contextos (*p.ex.*, o agressor sendo um estranho, conhecido ou amigo)^(70)^. Além disso, a violência de gênero é compreendida como um fenômeno social decorrente das relações patriarcais, ancorada em uma estrutura de poder desigual dos homens sobre as mulheres, que opera em todos espaços da sociedade^(76)^, incluindo universidades.

Esse entrelaçamento da violência de gênero com a subordinação social das mulheres não pode ser entendido nem reparado isoladamente em relação ao complexo mais amplo da violência na sociedade capitalista. Assim, é necessário articular lutas e ações para desmantelar as relações sociais e instituições que oprimem mulheres^(77)^. Isso indica a necessidade de pensar programas de prevenção que manejam a violência de forma mais abrangente, abordando mais de um tipo de violência no mesmo programa, considerando o contexto das relações sociais entre estudantes universitários.

Os achados sobre os vários **tipos de programa de prevenção** concordam com os resultados de uma meta-análise sobre programas para prevenir a violência em encontros universitários. Nessa análise, as estratégias usavam algum tipo de participação ativa (77%), como discussões em grupo (55%), apresentações didáticas e interativas (39%); havia outras atividades em grupo (36%) e usavam algum componente *online* (29%)^(70)^.

Discutir em grupo pode ser uma forma de conhecer e aproximar-se de pares, aumentando o senso de responsabilidade sobre o problema e o entendimento de que violência de gênero é um problema social nas universidades. Os resultados de um estudo qualitativo sobre violência sexual na Guatemala mostraram que os estudantes se sentem mais seguros quando se identificam e agem juntos como um grupo. A ação coletiva sinaliza às possíveis vítimas que elas não estão sozinhas, mas têm uma rede de apoio, além de mostrar aos possíveis agressores que suas ações não serão toleradas^(78)^. As sessões em grupo podem estimular o diálogo e a participação dos estudantes nas atividades. Condições para desenvolver o pensamento crítico são criadas por meio do diálogo, que envolve o movimento dialético entre o fazer e o pensar sobre o fazer, em um movimento que amplia a consciência^(79)^.

Além disso, a presente revisão identificou duas estratégias baseadas em jogos. Uma revisão sistemática apresentou o uso de jogos como ferramenta de aprendizado entre estudantes de enfermagem. Ela permitiu observar que o desejo de vencer a partida incentivou a participação e conferiu realismo à atividade, promovendo maior engajamento e diversão, tornando assim a aprendizagem mais prazerosa^(80)^.

Quanto à **forma de implementação dos programas** identificados na presente revisão, a maioria foi conduzida de modo presencial. Isso se alinha aos resultados de uma revisão de escopo que analisou estratégias de prevenção específica sobre violência por parceiro íntimo entre graduandos; intervenções administradas pessoalmente foram examinadas em 80% dos estudos incluídos^(81)^. O aspecto social da atividade presencial pode ajudar os estudantes a envolverem-se mais com o conteúdo dos programas, além de trazer a sensação de importância ao tópico abordado, pois os estudantes podem perceber a programação *online* como abstrata e impessoal. Os estudantes também perceberam que a independência facilitada por abordagens *online* prejudica a eficácia do programa^(68)^.

Quanto à forma de entrega, os resultados de uma meta- análise também apontaram que métodos digitais, tais como vídeos ou formatos exclusivamente *online*, são menos eficazes para desenvolver competências relacionadas ao papel de espectador. Eles sugerem que a interação presencial em um ambiente coletivo é mais eficiente para atingir esse objetivo^(70)^.

A **presença de facilitadores** foi identificada em 20 das 41 estratégias incluídas, sendo localizada só nas estratégias presenciais. Esse dado é um pouco menor que o relatado em uma meta-análise sobre programas para prevenir violência em encontros universitários, em que 61% das estratégias tinham facilitadores^(70)^. Uma meta-análise sobre a eficácia de programas de espectadores para prevenir violência sexual mostrou que diferença entre os efeitos de programas conduzidos por facilitadores e aqueles autodirigidos, tais como vídeos ou campanhas de posters, não foi significativa. Ambos formatos de programas tiveram efeitos positivos nas atitudes, crenças e comportamentos dos espectadores. Além disso, os efeitos nos programas ministrados por um facilitador não variaram se ele era ou não um colega da universidade^(67)^.

Na presente revisão, **a duração e o número de sessões** das estratégias variaram segundo seu formato, dificultando a síntese dos dados. Porém, foi possível observar uma predominância de sessões únicas, com duração variando entre minutos e horas para a maior parte das estratégias (exceto campanhas de *marketing* que tinham maior tempo de exposição). Isso se repetiu na meta- análise sobre programas para prevenir violência em encontros universitários, onde 77% das estratégias foram ministradas em uma única sessão, e as intervenções duraram de 18 min a 12 h (a maioria delas foi <2 h)^(70)^.

Um outro estudo de revisão mostrou que as intervenções presenciais requerem mais tempo que aquelas *online* para serem concluídas pelos participantes. Esse mesmo estudo apontou a dificuldade da avaliação crítica em estabelecer padrões sobre tempo, modo e frequência das intervenções, pois muitos estudos primários não relataram informações essenciais sobre a entrega da intervenção^(81)^. Cabe então considerar a evidência de que a duração do programa foi positivamente associada aos seus efeitos nas atitudes e crenças dos espectadores, *i.e*, quanto mais longo o programa, maior o efeito nas atitudes e crenças dos espectadores. Porém, essa associação não ocorreu para o comportamento dos espectadores^(67)^.

Ainda que esta revisão de escopo não tenha como objetivo avaliar os efeitos das intervenções, tais achados permitem problematizar sua duração e continuidade. Nesse sentido, universidades podem beneficiar-se da implementação de programas mais extensos ou de ações preventivas contínuas, incluindo abordagens multicomponentes. Além disso, a integração dos treinamentos de espectadores aos currículos dos cursos^(82)^ aliada à formação continuada baseada em evidências científicas para a comunidade^(83)^ são estratégias indicadas para construir um ambiente universitário seguro e justo.

Assim, destaca-se a importância dos **conteúdos abordados nos treinamentos**. A presente revisão de escopo identificou a predominância de temas relacionados a atitudes, competências e intervenções de espectadores; foram também identificados temas tais como mitos relacionados ao estupro e estereótipos de gênero; definições, conceitos e epidemiologia da violência; e consentimento e comunicação saudável. Os temas relacionados a valores, consciência, ética pessoal, recursos disponíveis, serviços, riscos, barreiras e obstáculos foram menos comuns.

Esses resultados concordam com aqueles de outros estudos: as revisões de conteúdos mais comuns para as estratégias concentraram-se no desenvolvimento de competências (81%) e formação de espectadores (74%), seguidas por discussões sobre tipos de comportamentos violentos (42%), prevalência de violência no namoro e/ou sexual (39%), definição de consentimento (39%), recursos disponíveis no *campus* (36%) e consequências da violência no namoro e/ou sexual (32%)^(70)^.

Além disso, uma revisão que investigou programas de intervenção de espectadores, com foco em prevenir a violência sexual em universidades, encontrou os seguintes temas de maior destaque: comportamento do espectador, probabilidade de intervir, eficácia, confiança e vontade de agir, bem como mudanças nas atitudes, crenças e normas^(84)^. A predominância de conteúdos voltados para atitudes, competências e intervenções de espectadores (bem como os mitos relacionados ao estupro e estereótipos de gênero) pode ser mais bem compreendida a partir dos próprios objetivos das estratégias de intervenção centradas no espectador, que visam capacitar os estudantes a desempenhar um papel ativo em reduzir a violência de gênero.

A segunda categoria de conteúdos identificada com mais frequência nos estudos foi relacionada a definições, conceitos e epidemiologia da violência. Algumas evidências indicam que os programas incluindo dados sobre a prevalência local de violência produziram maiores efeitos no sentimento de eficácia e nas intenções dos espectadores em relação à prevenção de violência no namoro. O estudo ainda sugeriu que o conhecimento sobre as altas taxas de violência no *campus* pode trazer mais consciência sobre a gravidade do problema e a necessidade de reduzir a prevalência, encorajando a intervenção dos participantes^(70)^. Isso indica que os programas para prevenir a violência de gênero podem beneficiar-se da inclusão de dados locais sobre a epidemiologia de violência em sua programação. Assim, a abordagem de definições culturalmente contextualizadas contribui para que os participantes possam reconhecer situações e comportamentos violentos.

Informações sobre recursos disponíveis e serviços para ajudar estudantes em situação de violência na universidade foram abordadas só em cinco estudos nesta revisão. Um dos estudos foi realizado com graduandos em três universidades e mostrou que quase metade deles desconhecia os recursos disponíveis em seu *campus* ^(85)^. Isso indica limitação nas estratégias de prevenção, pois falta de informação sobre serviços de apoio institucional pode restringir as respostas dos espectadores às situações de violência.

Por outro lado, evidências indicam que o conhecimento de estudantes sobre políticas e procedimentos da universidade relacionados a agressão sexual foi significativamente relacionado a suas intenções de se envolver em comportamentos e/ou atitudes pró-sociais de espectadores^(86)^. As universidades devem então formalizar políticas institucionais de prevenção à violência de gênero, promovendo acesso aos membros da comunidade acadêmica a recursos para defesa de seus direitos e informações sobre procedimentos de segurança em casos de violência^(82)^.

Conteúdos sobre riscos, barreiras e obstáculos à intervenção foram identificados só em quatro estudos. Porém, um estudo sobre violência doméstica e sexual identificou consequências adversas aos espectadores que intervieram em incidentes, incluindo relatos de assédio, ameaças e agressões físicas^(87)^.

Estatísticas detalhadas sobre a incidência de diferentes tipos de violência de gênero e a demografia de agressores e vítimas permitem adaptar os programas às necessidades específicas da comunidade universitária. Além disso, coleta e análise contínuas de dados são fundamentais para avaliar a eficácia das intervenções, focando especificidades e tipos de violência, fazendo então ajustes conforme necessário. Isso implica em analisar criticamente as estruturas sociais e econômicas que sustentam a violência, bem como em promover intervenções, buscando transformar essas estruturas e criar um ambiente acadêmico mais justo e seguro a todos.

Uma limitação deste estudo foi a impossibilidade de acessar todos dados sobre a caracterização de estratégias. Não foi possível identificar a duração das estratégias em um dos artigos e identificar o número de seções em outros. O(s) objetivo(s) específico(s) de cada treinamento não foi/foram sistematizado(s).

## CONCLUSÃO

Os treinamentos de espectadores para prevenir a violência de gênero em estudantes universitárias concentram-se majoritariamente em contextos norte-americanos, com foco predominante no tema da prevenção da violência sexual. Há diversidade de formatos de intervenção, incluindo sessões em grupo, campanhas de *marketing*, cursos *online*, *workshops* e jogos, com ênfase em ações presenciais e grupos mistos em relação ao gênero. A maioria das estratégias têm caráter pontual, sem integração a políticas institucionais. Nos treinamentos, os conteúdos mais abordados concentram-se em definições e conceitos de violência, mitos e estereótipos de gênero, consentimento e atitudes de espectadores. Há limitada exploração de barreiras à intervenção e de recursos institucionais nos conteúdos das estratégias, o que pode limitar o alcance das ações de prevenção da violência de gênero, representando uma lacuna nas estratégias identificadas.

Este estudo aponta para a necessidade de intervenções mais abrangentes, abordando diferentes formas de violência de gênero e realizadas continuamente. Futuras intervenções devem ampliar o escopo de conteúdos, incluindo riscos, barreiras e recursos institucionais, sendo incorporadas a políticas permanentes de prevenção em universidades. Essas medidas contribuem para construir ambientes acadêmicos mais seguros, equitativos e comprometidos em prevenir a violência de gênero.

## Data Availability

As estratégias de busca usadas em cada fonte de informação estão disponíveis acessando o *link* seguinte: (https://osf.io/x4pnf/files/hkvc6).

# Translation: Capacitación de espectadores para prevenir la violencia de género contra estudiantes universitarias: una revisión del alcance*

**DOI:** 10.1590/1980-220X-REEUSP-2025-00231es

**Authors:** Taís Tasqueto Tassinari, Letícia de Mello Padoin, Laís Alves Molina, Cristiane Cardoso de Paula, Stela Maris de Mello Padoin

**Affiliations:** 1 Prefeitura Municipal de Penha, Departamento de Vigilância Sanitária, Penha, SC, Brasil.; 2 Universidade Federal de Santa Catarina, Programa de Pós-graduação em Serviço Social, Florianópolis, SC, Brasil.; 3 Universidade Franciscana, Residência Profissional em Enfermagem Obstétrica, Santa Maria, RS, Brasil.; 4 Universidade Federal de Santa Maria, Programa de Pós-graduação em Enfermagem, Santa Maria, RS, Brasil.

**Keywords:** Prevención, Universidades, Violencia de Género, Revisión de Alcance, Mujeres

## Abstract

**Objetivo:**

Mapear las características de la capacitación para espectadores con el fin de prevenir la violencia de género contra las estudiantes en un contexto universitario.

**Método:**

Revisión del alcance utilizando el método JBI. La búsqueda se realizó en marzo de 2025 en MEDLINE/PubMed, WoS, BVS, EBSCO, EMBASE, Scopus, PsycInfo/APA y SciELO, independientemente del idioma, con un período de referencia que abarcó desde 2016. La selección y extracción fueron doblemente independientes, con un análisis descriptivo de la caracterización y un enfoque inductivo del contenido de la formación.

**Resultados:**

En 46 estudios incluidos, se identificaron 9 tipos de capacitación que tenían como objetivo prevenir la violencia sexual mediante un enfoque mixto. El contenido de la capacitación se clasificó en: definiciones, conceptos y epidemiología de la violencia; mitos sobre la violación y estereotipos de género; consentimiento y comunicación saludable; actitudes, comportamientos e intervenciones de los espectadores; recursos y servicios disponibles; valores, concienciación y ética personal; riesgos, barreras y obstáculos para la intervención.

**Conclusión:**

La deficiencia radica en la concentración temática de los estudios, centrados en la violencia sexual, con una exploración limitada de los riesgos, las barreras para la intervención y los recursos institucionales en el contenido de las estrategias.

## INTRODUCCIÓN

La violencia de género es un problema global observado en contextos sociales y económicos arraigados en relaciones de poder desiguales, con una estructura patriarcal, que afecta principalmente a las mujeres^(1)^. Ellas son las más perjudicadas en el contexto universitario y la prevalencia de la violencia sexual entre los estudiantes puede ser mayor que en la población general^(2)^. Un estudio de revisión con estudiantes universitarios encontró tasas de prevalencia que oscilaban entre el 1,3 % y el 88 %, con una amplia variación según el tipo de violencia, la herramienta de Recolección de datos y la muestra de los estudios^(3)^.

La violencia de género entre las estudiantes universitarias se manifiesta de diversas formas; entre los agresores se encuentran sus parejas y exparejas, compañeros y otros estudiantes, profesores y personal de las universidades^(4)^. La violencia que sufren está presente en su vida cotidiana y ocurre en entornos domésticos (como sus hogares y los de los agresores), lugares de la universidad (como aulas y espacios comunes del campus) y fiestas^(5)^.

La violencia contra las universitarias afecta gravemente su salud física y mental, así como surendimiento académico. La violencia por parte de parejas íntimas puede desencadenar episodios de depresión y estrés postraumático, cuya gravedad está significativamente correlacionada con la intensidad de la violencia^(6)^. Además de los síntomas de ansiedad y comportamiento suicida debido a la violencia sufrida, los resultados de una revisión sistemática mostraron que la violencia sexual se asoció con promedios de calificaciones más bajos, ausentismo y deserción escolar^(7)^.

La normalización y trivialización de la violencia en los espacios educativos es un agravante para las mujeres que enfrentan esta situación. Con frecuencia, las experiencias de violencia de género son silenciadas y los discursos de las víctimas son invalidados para preservar la imagen institucional^(8)^. El proceso de «traición institucional» está relacionado con las estructuras sociales de poder y opresión, lo que contribuye a perpetuar la violencia. Esto se manifiesta en varios niveles institucionales en forma de políticas que dificultan las denuncias de violencia, burocratizan los procesos, se niegan a apartar o destituir a los agresores de alto *estatus* y en la falta de compromiso para promover cambios sistémicos y duraderos en las universidades^(9)^.

Para comprender mejor las posibilidades de enfrentar la violencia de género en el ámbito universitario, es relevante identificar estrategias preventivas que promuevan la participación social para la rendición de cuentas y la transformación cultural de los roles de género. En este sentido, la literatura apunta a intervenciones con capacitación de espectadores; estas parten del supuesto de que los líderes pueden fomentar cambios sociales en las comunidades para responder a un problema de salud pública, por ejemplo, la violencia de género. El término «espectadores» se refiere a personas que presenciaron el antes, durante o después de un evento violento. Así, el entrenamiento de espectadores tiene como objetivo capacitar a las personas para que puedan intervenir en actos violentos o normas sociales que perpetúan la violencia, buscando prevenir su ocurrencia^(10)^.

Algunos factores pueden promover comportamientos adecuados en los espectadores para que sus acciones sean eficaces, lo que resulta en la prevención de la violencia. Algunos ejemplos son los siguientes: contenidos y mensajes que ayudan a los espectadores a identificar situaciones de violencia; desarrollo de habilidades y práctica de los espectadores para aumentar su sentido de eficacia utilizando modelos que muestran intervenciones que ellos pueden reproducir; y un enfoque de intervención que respeta y considera la propia seguridad del espectador para que los beneficios de intervenir superen las barreras^(11)^.

Así, la capacitación de los espectadores fortalece las habilidades para reducir el riesgo de violencia, reconociendo situaciones que pueden volverse violentas, interviniendo con seguridad y eficacia, y manifestándose en contra de actitudes que estimulan o toleran comportamientos violentos. La capacitación tiene como objetivo reducir la violencia modificando las normas sociales y promoviendo cambios en las actitudes y comportamientos de los espectadores, incluyendo sus creencias e intenciones sobre cómo actuar. De este modo, la pasividad frente a la violencia puede desincentivarse como estrategia para remodelar y estimular la responsabilidad social, previniendo la violencia de género^(12)^.

La literatura muestra que existen varios estudios sobre el entrenamiento de espectadores, que indican distintos formatos de intervención^(13,14,15)^. Sin embargo, el esfuerzo por comprender y sistematizar estas estrategias de prevención sigue siendo fragmentado. Los datos son dispersos en cuanto a los métodos y sus características, tales como la duración, el contenido, el formato y el contexto de aplicación de las intervenciones. Tras identificar esta laguna, surgió la necesidad de mapear la evidencia sobre los entrenamientos para prevenir la violencia de género, lo que señaló la necesidad de una revisión.

Además, se realizó una búsqueda preliminar de estudios sobre el tema y no se identificaron revisiones de alcance ni protocolos en curso en el *International Prospective Register of Systematic Reviews* (PROSPERO)*, Open Science Framework* (OSF)*, Medical Literature Analysis and Retrieval System Online* (MEDLINE), *la Base de Datos Cochrane de Revisiones Sistemáticas* y JBI *Evidence Synthesis*. Esta situación confirma la laguna de conocimiento sobre el tema y la necesidad de desarrollar una revisión de alcance. Por lo tanto, el objetivo de esta revisión fue mapear las características de los entrenamientos de espectadores para prevenir la violencia de género en mujeres estudiantes en el contexto universitario.

## MÉTODO

### Diseño del Estudio

Esta revisión de alcance se llevó a cabo según el método del JBI^(16)^ y se informó de acuerdo con los *Preferred Reporting Items for Systematic reviews and Meta-Analyses extension or Scoping Reviews* (PRISMA-ScR)^(17)^. El protocolo se registró en el *Open Science Framework* (https://doi.org/10.17605/OSF.IO/4NK9U).

### Identificación de la Pregunta de la Revisión

La pregunta de la revisión («¿Cuáles son las características de los entrenamientos de espectadores para prevenir la violencia de género en mujeres estudiantes en el contexto universitario?») se estructuró a partir del acrónimo PCC; que indica población (**P**: estudiantes universitarios), concepto (**C**: prevención de la violencia de género entre las estudiantes universitarias, mediante la formación de espectadores) y contexto (**C**: *campus* universitarios).

A partir de esta pregunta, se elaboraron las preguntas secundarias: ¿Cuáles son los tipos de intervención descritos en la literatura? ¿Cuál es la forma de implementación de las intervenciones? ¿Las intervenciones cuentan o no con la presencia de un mediador? ¿Cuál es la duración de las intervenciones? ¿Cuántas sesiones o etapas tienen las intervenciones? ¿Cuál es el contenido de las intervenciones?

### Criterios de Elegibilidad

Participantes: fueron elegibles los estudios realizados con estudiantes universitarios (de ambos sexos y géneros, sin restricción de edad) matriculados en cursos de pregrado o posgrado. La revisión incluyó muestras (tanto generales como específicas) de estudiantes universitarios, como estudios solo con mujeres, estudiantes de pregrado, de posgrado o miembros de hermandades y/o fraternidades.

Concepto: se consideraron elegibles los estudios que describían la capacitación de espectadores orientada a prevenir la violencia de género contra las universitarias (cisgénero y transgénero), abarcando la violencia de pareja, física, sexual, psicológica, moral o patrimonial. Las intervenciones podían presentarse en diversos formatos (programas educativos, videos, capacitaciones en línea, campañas, clases o charlas), sin limitación en cuanto a la duración o el número de sesiones, y podían incluir o no facilitadores. Se excluyeron los estudios centrados en estrategias profesionales de prevención de la violencia, autodefensa, y aquellos que combinaban la prevención de la violencia con la prevención de infecciones de transmisión sexual (ITS) o el consumo abusivo de alcohol, debido a la complejidad de sintetizar estos temas.

Contexto: fueron elegibles los estudios realizados en el *campus* de universidades públicas o privadas de cualquier país, y a través de plataformas digitales. Se excluyeron los estudios sobre intervenciones realizadas fuera de las instituciones educativas, aunque estuvieran respaldadas por estas.

Tipo de estudio: se consideraron elegibles los diseños de estudios experimentales y cuasi-experimentales, incluyendo estudios controlados (antes y después) y estudios de series temporales interrumpidas. Esta revisión también consideró estudios observacionales, cualitativos y mixtos. Tras una búsqueda exploratoria, se identificó que la producción de literatura gris presentaba limitaciones en cuanto a la presentación de las capacitaciones y la descripción metodológica, lo que comprometía la consistencia en la claridad, la selección y la exhaustividad para extraer datos. Así, por ejemplo, se excluyeron editoriales, artículos de opinión o reflexión, boletines epidemiológicos, legislaciones estatales y federales, guías, folletos y notas técnicas.

En cuanto al año de publicación de los estudios, se consideró como marco temporal el Día Internacional de las Mujeres y las Niñas en la Ciencia, el 11 de febrero, aprobado por la Asamblea de las Naciones Unidas el 22 de diciembre de 2015. Aunque el marco es simbólico y político, su adopción como criterio metodológico se justifica por señalar la necesidad de que el compromiso global de cambio de paradigma siga vigente para garantizar la igualdad de género en los entornos de investigación. Por lo tanto, fueron elegibles para esta revisión los estudios publicados a partir de 2016, alineando el nuevo paradigma de protección de las estudiantes en el espacio académico con el levantamiento bibliográfico de una acción práctica, como la capacitación de espectadores, con un período de movilización institucional para enfrentar la violencia en el contexto de la enseñanza y la investigación.

### Estrategia de Búsqueda y Fuentes de Información

Siguiendo las recomendaciones de la JBI, la estrategia de búsqueda se llevó a cabo en tres etapas. La etapa 1 consistió en una búsqueda limitada a la base de datos *Medical Literature Analysis and Retrieval System Online* (MEDLINE/PubMed), donde se analizaron las palabras clave en los títulos y resúmenes de los artículos identificados.

Tras este mapeo de términos, se desarrollaron las estrategias de búsqueda en las demás fuentes de información (etapa 2).

Las fuentes de información consultadas para recuperar estudios incluyen 8 bases de datos y bibliotecas virtuales: *Medical Literature Analysis and Retrieval System Online* (MEDLINE/PubMed); *Web of Science Core Collection* (WoS); Biblioteca Virtual en Salud (BVS); EBSCO; EMBASE; *SciVerse Scopus* (Scopus); *PsycInfo* (APA) y *Scientific Electronic Library Online* (SciELO). Se incluyeron estudios publicados en cualquier idioma. Todas las búsquedas se realizaron en marzo de 2023 y se actualizaron en febrero-marzo de 2025. La etapa 3 consistió en una búsqueda en la lista de referencias, pero no se incluyó ninguna referencia en esta etapa. Todas las etapas se llevaron a cabo con la colaboración de una bibliotecaria especialista en estudios de revisión en el área de la salud.

### Selección de los Estudios

Las referencias se agruparon y se cargaron en el *software Endnote* 20/2020 (*Clarivate Analytics*; PA, EE. UU.); tras eliminar los duplicados, se exportaron al sistema Rayyan (*Qatar Computing Research Institute*, QCRI)^(18)^. Dos revisoras seleccionaron los títulos y resúmenes para su evaluación (modo doble independiente) de acuerdo con los criterios de elegibilidad. Los estudios potencialmente relevantes se recuperaron en su totalidad y fueron evaluados detalladamente por las revisoras (manteniendo la independencia del proceso) según los criterios de elegibilidad. Se registraron y comunicaron los motivos de exclusión de los estudios de texto completo. En cada etapa del proceso de selección, las discrepancias que surgieron entre las revisoras se resolvieron mediante el debate con una tercera revisora, experta en el tema de la violencia de género. Los resultados de la búsqueda y el proceso de selección de los estudios se comunicaron íntegramente y se presentaron en un diagrama de flujo^(17)^.

### Extracción de Datos y Presentación de los Resultados

La extracción de la evidencia fue multifásica. Para la fase de caracterización de los estudios se utilizó la herramienta estandarizada: país, año, revista, población, objetivo y método de los estudios^(16)^. Para la fase de mapeo del concepto, se extrajeron los siguientes datos: tipo de intervención (presentación de pósteres, video, curso *en línea*, juego educativo, etc.); contenido de la intervención; características de las intervenciones (duración, presencia o no de un mediador, número de sesiones y/o etapas, tipo específico de violencia que se busca prevenir) y forma de implementación de la intervención (presencial o *en línea*). Cabe destacar que, en siete estudios, se identificaron dos intervenciones diferentes y una misma intervención se evidenció en estudios distintos, lo que dio lugar a que el número de estudios incluidos fuera diferente del total de estrategias mapeadas.

### Análisis de los Datos

Se utilizó un análisis descriptivo para caracterizar los estudios primarios y las características de las capacitaciones, utilizando la frecuencia simple. La presentación de estos resultados (incluidas figuras y cuadros) se organizó gráficamente, acompañada de su descripción. Para analizar el contenido de las capacitaciones, se utilizó el enfoque inductivo de análisis de contenido cualitativo básico; este es un enfoque descriptivo de análisis que implica la codificación abierta para asignar características a categorías generales^(19)^. Este análisis se llevó a cabo en tres fases: 1) preparación: familiarización con los datos mediante la lectura de las fuentes de evidencia para identificar el contenido de las estrategias de capacitación de espectadores, con el objetivo de prevenir la violencia de género en mujeres estudiantes en el contexto universitario; 2) organización: identificación de los temas de cada estrategia y codificación de los contenidos por similitud en categorías; 3) informe: presentación descriptiva y gráfica de los resultados^(20)^.

No se llevó a cabo una evaluación de la calidad metodológica, teniendo en cuenta la distinción entre revisiones sistemáticas y de alcance, en las que las primeras deben incluir una evaluación crítica o del riesgo de sesgo para recomendar la implementación de los resultados sintetizados en políticas o prácticas. En cambio, no es obligatorio que las revisiones de alcance evalúen la calidad de la evidencia incluida, sino que ofrezcan una visión general de la frontera del conocimiento para recomendar la descripción de la evidencia mapeada con el fin de implementar investigaciones sobre las brechas de conocimiento^(16)^.

## RESULTADOS

Las referencias seleccionadas se agruparon y cargaron en el *software* de gestión de referencias Endnote 20/2020. Se eliminaron los duplicados; a continuación, las referencias restantes se exportaron a la plataforma en línea Rayyan, donde se llevó a cabo la lectura de títulos y resúmenes. Se seleccionaron un total de 127 estudios para su lectura completa aplicando los criterios de elegibilidad. De estos, 81 fueron excluidos por no cumplir con los criterios de elegibilidad; así, 46 estudios conformaron el cuerpo de esta revisión (Figura 1).

**Figura 1:** Diagrama de flujo Preferred Reporting Items for Systematic Reviews and Meta-analyses extension for scoping review (PRISMA-ScR – Santa Maria, RS, Brasil, 2025).

En el Cuadro 1 se muestran el país donde se realizaron los estudios, el año de publicación, el objetivo, el tipo de estudio, el número y el género de los participantes. En los estudios primarios, no se identificó uniformidad en la forma de presentación de la información relativa al género de los participantes. Su extracción siguió la terminología empleada en cada estudio. En el Cuadro 1, esta información se sintetizó y representó mediante símbolos, utilizados específicamente con fines ilustrativos y de organización del cuadro.

**Cuadro 1:** Características de los estudios incluidos – Santa María, RS, Brasil, 2025.

País y año	Objetivo	Tipo de estudio y participantes
EE. UU., 2017^(21)^	Investigar la eficacia de *The Women’s Program*, un programa de sensibilización sobre la violencia sexual dirigido a mujeres.	Casi experimental. n: 103 ♀
EE. UU., 2018^(22)^	Examinar cómo funcionaron dos métodos de prevención, por separado y de forma conjunta, para promover un cambio de actitud respecto a la violencia sexual entre los estudiantes universitarios.	Casi experimental. n:948 ♀ ♂
EE. UU., 2019^(23)^	Proporcionar la descripción y los datos de evaluación de un programa para prevenir la violencia sexual, impartido a estudiantes de primer curso durante el primer semestre en una gran universidad pública.	Casi experimental. n:2.305 ♀ ♂
EE. UU., 2016^(24)^	Analizar si una intervención breve y fácil de implementar en toda la universidad podría mejorar los comportamientos, las intenciones, las actitudes, la eficacia y las normas sociales de los espectadores para prevenir la violencia en el noviazgo, y comprender qué variables estaban más estrechamente relacionadas con los comportamientos de los espectadores.	Casi experimental. n:288 ♀ ♂
EE. UU., 2021^(25)^	Analizar la eficacia de una intervención denominada *programa bystander plus*.	Casi experimental. n:81 ♀ ♂
EE. UU., 2022^(13)^	Describir el desarrollo y la evaluación de un programa de prevención primaria de la violencia sexual y en el noviazgo (*Red Flag Campaign*) ampliamente difundido.	Casi experimental. n:203 ♀ ♂
EE. UU., 2016^(26)^	Examinar los efectos de *Green Dot* en las tasas de victimización y violencia entre los estudiantes de primer curso con datos recopilados durante 4 años (2010–2013).	Casi experimental. n:7.111 ♀ ♂
EE. UU., 2018^(27)^	Evaluar en qué medida los talleres del *Programa de Prevención de la Violencia contra las Mujeres* reducen la aceptación del mito de la violación por parte de los estudiantes, aumentan su conocimiento y comprensión sobre la política de consentimiento afirmativo de la universidad y mejoran su confianza a la hora de interpretar las señales relacionadas con el consentimiento sexual.	Experimental. n:1.957 ♀ ♂
Australia 2021^(28)^	Aumentar la sensibilización y los conocimientos previos de los estudiantes de ciencias de la salud sobre el contexto de la violencia de género y lograr que los participantes aprendan estrategias de intervención como testigos para actuar de forma eficaz y reducir la violencia.	Casi experimental. Estudio piloto. n: 21 ♀ ♂
EE. UU., 2021^(29)^	Evaluar el programa *Wingman* 101, destinado a prevenir la violencia sexual mediante la intervención de los testigos, diseñado para deportistas universitarios masculinos y miembros de fraternidades de.	Casi experimental. n:80 ♂
EE. UU., 2020^(14)^	Examinar la eficacia del programa *Safe Sisters* para la intervención en espectadores sobre la agresión sexual, dirigido a miembros de hermandades universitarias.	Experimental. n:139 ♀
EE. UU., 2017^(30)^	Identificar los factores predictivos del comportamiento de los espectadores y la asertividad sexual entre las estudiantes de las hermandades. Evaluar los cambios en la aceptación del mito de la violación, la autoeficacia de los espectadores y la asertividad sexual dos semanas después de que se impartiera el *programa The Women’s Program* a las miembros de las hermandades, y describir los comportamientos de los espectadores durante las dos semanas posteriores a la intervención.	Casi experimental. n: 141♀
Australia 2024^(15)^	Evaluar un módulo educativo en línea sobre la prevención y la respuesta a la violencia sexual diseñado e implementado en una universidad australiana.	Mixto. n:113♀ ♂O
EE. UU., 2018^(31)^	Examinar la eficacia de una formación para la intervención de espectadores *Green Dot* adaptada a los residentes de medicina.	Casi experimental. n: 24♀ ♂
EE. UU., 2017^(32)^	Evaluar un programa de prevención centrado en diversos aspectos de la agresión sexual, incluyendo contenidos sobre el consentimiento sexual, la intervención de los testigos, la denuncia de la violencia sexual y de los incidentes de agresión, la búsqueda de ayuda tras un incidente y el apoyo a las víctimas de agresión sexual.	Casi experimental. n:146♀ ♂
EE. UU., 2018^(33)^	Adaptar, replicar y evaluar la generalización del programa *Bringing the Bystander* ^TM^ en una facultad jesuita privada de artes liberales de Massachusetts.	Casi experimental. n:164♀ ♂
EE. UU., 2021^(34)^	Evaluar si un curso semestral para estudiantes de primer curso influyó en los conocimientos, las actitudes y las intenciones de comportamiento sobre género, sexualidad y violencia sexual.	Casi experimental. n:109♀ ♂
EE. UU., 2016^(35)^	Informar sobre dos ensayos clínicos aleatorizados que evalúan el programa *TakeCARE* para espectadores de vídeo, diseñado para prevenir la violencia sexual en los *campus* universitarios.	Experimental. Estudio 1: n:213 ♀ ♂ Estudio 2: n:211 ♀ ♂
EE. UU., 2016^(36)^	Presentar la evaluación de un nuevo procedimiento desarrollado para medir el comportamiento de los espectadores y prevenir la violencia sexual.	Casi experimental. n: 91 ♀ ♂
EE. UU., 2020^(37)^	Replicar y ampliar los hallazgos anteriores sobre los efectos del vídeo *TakeCARE* en el comportamiento de los espectadores, probando dos métodos de presentación del vídeo y examinando los factores moderadores de sus efectos en el comportamiento de los espectadores.	Experimental. n:557 ♀ ♂
Australia 2020^(38)^	Ofrecer una visión general del proceso y la implementación, así como de los retos y los éxitos encontrados en una universidad australiana al adoptar el taller acreditado *Sex, Safety and* Respect, basado en la evidencia.	Relato de experiencia. n:118♀ ♂
EE. UU., 2020^(39)^	Describir una campaña de intervención dirigida a los espectadores puesta en marcha en una universidad históricamente negra del sureste de EE. UU.	Cualitativo. n:406♀ ♂
EE. UU., 2021^(40)^	Evaluar la eficacia de una innovadora campaña de *marketing* de normas sociales llevada a cabo durante 5 años, destinada a involucrar a los hombres en la prevención de la violencia sexual en un gran *campus* universitario público del sureste de EE. UU.	Serie temporal. n: 4.158 ♂
India 2022^(41)^	Evaluar la eficacia del programa *RISE* para prevenir la violencia sexual entre las estudiantes universitarias en la India.	Casi experimental. n:245 ♀
EE. UU., 2021^(42)^	Evaluar la eficacia de la intervención *en línea STOP Dating Violence*, desarrollada para educar a los estudiantes universitarios sobre la violencia en el noviazgo y las intervenciones de los testigos en *los campus* universitarios.	Experimental. n:317♀ ♂
EE. UU., 2018^(43)^	Comparar la eficacia de un programa de educación para testigos (90 min) destinado a prevenir la violencia en el noviazgo con la de un programa tradicional de sensibilización sobre el tema, incluyendo un grupo de control (sin intervención), en cuanto al cambio de actitudes, creencias, eficacia percibida, intenciones y comportamientos autoinformados en estudiantes universitarios.	Casi experimental. n: 1.001♀ ♂
EE. UU., 2019^(44)^	Describir la creación de dos videojuegos para enseñar a los estudiantes universitarios habilidades de intervención como espectadores en situaciones de violencia sexual, de pareja y acoso.	Casi experimental. n:305 ♀ ♂
EE. UU., 2021^(45)^	Evaluar la eficacia de dos juegos de prevención *en línea* dirigidos a estudiantes universitarios: (1) preguntas y respuestas y (2) un juego de aventuras que enseñan habilidades de intervención a los espectadores para prevenir la violencia sexual.	Experimental. n:227 ♀ ♂
EE. UU., 2018^(46)^	Evaluar la eficacia del taller *Escalation* para prevenir el abuso en las relaciones de pareja con una muestra de estudiantes universitarios.	Experimental. n:85♀ ♂
EE. UU., 2018^(47)^	Examinar el proceso mediante el cual un programa de prevención de la violencia sexual basado en la web (*RealConsent*) previene la violencia sexual y mejora el comportamiento de los espectadores.	Experimental. n:743♂
Nueva Zelanda 2021^(48)^	Presentar los resultados cuantitativos y cualitativos de una prueba piloto del taller Bring in *The Bystander*, desarrollado para abordar el problema de la violencia sexual en *los campus* universitarios adoptando un enfoque basado en los valores comunitarios.	Mixto. CUANT+CAL. Estudio piloto. n:167♀ ♂
EE. UU., 2018^(49)^	Presentar la puesta en marcha y la evaluación de una campaña basada en la teoría para promover la intervención activa de los espectadores.	Casi experimental. n: 1.505♀ ♂
EE. UU., 2016^(50)^	Evaluar el recuerdo, la reacción y la comprensión de una breve campaña con carteles en *el campus* que promovía el consentimiento en las relaciones sexuales, y determinar si la exposición a la campaña se asociaba con la participación posterior en actividades relacionadas con la educación, la sensibilización y la prevención de la violencia sexual.	Mixto. n: 628 ♀ ♂
China 2019^(51)^	Evaluar la viabilidad del *programa Dating Compassion, Assessment, reFerral, and Education* (CAFE) *Ambassador* en China.	Casi experimental. n:85 ♀ ♂
EE. UU., 2022^(52)^	Evaluar una breve intervención *en línea* para reducir la agresión sexual y mejorar los comportamientos prosociales de los espectadores entre estudiantes universitarios heterosexuales varones.	Casi experimental. n:241♂
Vietnam 2022^(53)^	Adaptar el programa *RealConsent* para prevenir la violencia sexual (basado en la teoría y en la web) para estudiantes universitarios en Vietnam y evaluar el impacto del programa adaptado *GlobalConsent* en este contexto.	Experimental. n:730♂
Vietnam 2023^(54)^	Evaluar el impacto del programa *GlobalConsent* en el comportamiento sexualmente violento y en el comportamiento prosocial de los espectadores entre los hombres universitarios de Vietnam.	Experimental. n:793♂
EE. UU., 2021^(55)^	Examinar el impacto a corto plazo de un curso para prevenir la violencia sexual muy utilizado entre los estudiantes universitarios como enfoque preventivo a nivel poblacional.	Casi experimental. n:167 424 ♀ ♂TO
Inglaterra 2025^(56)^	Explorar la viabilidad de implementar *The Intervention* Initiative en un entorno universitario del norte de Inglaterra.	Mixto. n:21 ♀ ♂ (grupos focales y entrevistas)
EE. UU., 2023^(57)^	Describir el impacto de *myPlan* (herramienta de apoyo a la toma de decisiones en materia de seguridad personalizada) en amigos preocupados (estudiantes universitarios de cualquier género de entre 18 y 24 años), que se identifican a sí mismos como personas que tienen una amiga que ha sufrido violencia por parte de su pareja en los últimos 6 meses.	Experimental. n:293 ♀ ♂
EE. UU., 2024^(58)^	Examinar los efectos de una intervención de sensibilización sobre el comportamiento del espectador entre estudiantes universitarios que han completado un programa de formación para espectadores.	Mixto. Estudio 1: n:13♀ ♂NB Estudio 2: n: 38 ♀ ♂NB
Canadá 2023^(59)^	Utilizar técnicas metaanalíticas para explorar los datos de un programa presencial destinado a prevenir la violencia en las relaciones de pareja (impartido antes de la COVID-19).	Casi experimental. Serie temporal. n:244 ♀ ♂NB,T
EE. UU., 2024^(60)^	Examinar el impacto de un programa autodidáctico *en línea* centrado en la intervención de los testigos ante la violencia doméstica.	Casi experimental. n:293 ♀ ♂
EE. UU., 2023^(61)^	Describir el desarrollo del manual *SPoRT* (intervención para la prevención) y su evaluación inicial en cuanto a viabilidad y aceptabilidad.	Mixto. n:30 ♀ ♂
India 2024^(62)^	Investigar los efectos a corto plazo de los módulos *RISE-ON* en los conocimientos y las actitudes de los participantes.	Casi experimental. n:244♀
EE. UU., 2023^(63)^	Desarrollar y llevar a cabo una evaluación preliminar de la nueva aplicación digital *Make a Change* para prevenir la violencia sexual en la universidad entre los estudiantes de 2.º a 4.º curso.	Mixto. n:105♀ ♂

Leyenda: ♀ mujeres;

♂ hombres;

NB: no binarios; T: transgénero; O: otros géneros; GF: grupos focales.

En cuanto a los países de publicación, los estudios se realizaron principalmente en los Estados Unidos de América (n = 35), seguidos de Australia (n = 3), India (n = 2), Vietnam (n = 2), Nueva Zelanda (n = 1), China (n = 1), Canadá (n = 1) e Inglaterra (n = 1). De los 46 estudios incluidos, 34 presentaron estrategias dirigidas a grupos mixtos en cuanto al género, 7 solo para hombres y 5 solo para mujeres.

En cuanto al **tipo específico de violencia**, los estudios se centraron en prevenir la violencia sexual (34), la violencia doméstica en las relaciones y por parte de la pareja íntima (14), y la violencia interpersonal y de género contra las mujeres (abordadas de forma aislada o conjunta; 4).

Entre los 46 estudios incluidos, se identificaron 41 estrategias de capacitación (en algunos casos, un mismo estudio presentó evidencia sobre dos intervenciones diferentes y algunas intervenciones aparecieron más de una vez en estudios distintos). Dichas **estrategias se clasificaron en nueve tipos**: sesiones grupales (12)^(14,22,23,25,26,29,31,33,39,41,43,48,51,56,58,61)^, presentaciones interactivas y videos (5)^(21,24,30,35,37,42,52)^, campañas de *marketing* (6)^(13,22,24,40,49,50)^, cursos, módulos y herramientas educativas (8)^(15,34,47,53,54,55,59,60,62)^, talleres (4)^(27,28,38,46)^, videojuegos (2)^(44,45)^, aplicaciones (2)^(57,63)^, simulación en realidad virtual (1)^(36)^ y minirrevista (1)^(32)^ (Figura 2).

**Figura 2:** Identificación de las estrategias para prevenir la violencia de género, clasificadas por tipo – Santa Maria, RS, Brasil, 2025.

En cuanto a la clasificación sobre la **implementación de las 41 estrategias,** estas fueron presenciales (25)^(14,21,22,23,24,25,26,27,28,29,30,31,32,33,34,38,39,40,41,43,46,48,50,51,56,58,59,61)^, digitales o en línea (11)^(15,42,44,45,47,53,54,55,57,60,62,63)^ y mixtas, que combinaban acciones presenciales y elementos digitales (5)^(13,35,36,37,49,52)^.

La **presencia de facilitadores o mediadores** se identificó específicamente en estrategias impartidas de forma presencial, incluyendo estrategias de sesiones grupales (11)^(14,22,23,25,26,29,31,33,39,41,43,48,51,56,58,61)^, talleres (4)^(27,28,38,46)^, presentaciones interactivas y videos (2)^(21,24,30)^ y cursos, módulos o herramientas educativas (2)^(34,59)^. En total, 20 estrategias informaron la presencia de facilitadores o mediadores.

La **duración y el número de sesiones o presentaciones** variaron según el tipo de estrategia. Las sesiones grupales variaron de 1 a 8 horas en sesiones únicas^(14,23,31,33,43,56,58)^, y de dos^(25,30,48)^, tres^(51)^ o cuatro sesiones^(61)^. Dos estudios no informaron el número de sesiones^(26,41)^. Todos los talleres fueron sesiones únicas, con una duración de 40 minutos hasta un turno^(27,28,38,46)^. Los videos y las presentaciones interactivas fueron presentaciones únicas que variaron de 24 a 90 minutos^(21,24,30,35,37,42,52)^. Las campañas de *marketing* permanecieron expuestas durante períodos variables (de una semana a cinco años), lo que permitió numerosas visualizaciones^(13,22,24,40,49,50)^. Los cursos, módulos y herramientas educativas tuvieron una duración variable (de 45 minutos a un semestre), en una sola presentación^(15,55,62)^ o divididos en módulos^(34,47,53,54,59)^. En uno de los estudios no se encontró esta información^(60)^. Los videojuegos tuvieron una duración de 45 a 60 minutos^(44,45)^. Las minirrevistas se presentaron en cinco volúmenes (ocho páginas cada uno), entregados por correo, lo que permitió múltiples visualizaciones^(32)^. La simulación en realidad virtual se dividió en siete simulaciones, con una duración de 2 a 4 minutos cada una^(36)^. Las dos aplicaciones móviles tenían una duración total de 30 minutos^(63)^ y acceso ilimitado durante un año^(57)^.

En cuanto al **contenido de las estrategias**, se identificaron siete categorías: 1. Definiciones, conceptos y epidemiología de la violencia (n = 36); 2. Mitos relacionados con la violación y estereotipos de género (n = 25); 3. Consentimiento y comunicación sana (n = 26); 4. Actitudes, comportamientos e intenciones de los espectadores (n = 41); 5. Recursos disponibles y servicios (n = 5); 6. Valores, conciencia y ética personal (n = 10); y 7. Riesgos, barreras y obstáculos para la intervención (n = 4) (Figura 3).

**Figura 3:** Distribución cuantitativa de los estudios identificados por categoría de contenido – Santa Maria, RS, Brasil, 2025.

## DISCUSIÓN

El **predominio geográfico** de los estudios seleccionados fue de los EE. UU. (76 % del total). Este dato concuerda con otras revisiones sistemáticas sobre la prevalencia de la agresión sexual y las intervenciones educativas sobre la violencia de género, ya que ambas contenían aproximadamente el 73 % de sus estudios primarios procedentes de los EE. UU.^(64,65)^. Esto puede entenderse como el resultado de políticas federales, como el *Título IX*, que obliga a las universidades financiadas con fondos federales a desarrollar estrategias de prevención de la violencia, además de prohibir la discriminación sexual en programas y actividades educativas^(66)^. Dada la cantidad de estudios, se puede inferir que la existencia de la legislación también estimula la producción de datos sobre la prevalencia de la violencia en las universidades, así como estrategias de prevención.

En cuanto a los participantes en los estudios, hubo un predominio de estrategias dirigidas a grupos mixtos en cuanto al género. En este sentido, una revisión sistemática con metaanálisis evaluó la eficacia de los programas para espectadores que abordan la violencia sexual en los *campus* universitarios y no encontró diferencias entre los efectos cuando se dirigían a grupos mixtos o del mismo sexo. Los efectos se evaluaron para dos resultados: actitudes y/o creencias y comportamientos de los espectadores^(67)^.

Las estrategias dirigidas a grupos mixtos también se alinean con los deseos de los estudiantes universitarios, según los resultados de un estudio sobre sus preferencias en relación con las estrategias de prevención de la violencia en el *campus*. Los estudiantes perciben los programas dirigidos a grupos mixtos como más inclusivos en relación con el género y la diversidad sexual, además de que disipan los estereotipos sobre los roles que desempeñan hombres y mujeres en un incidente de agresión sexual^(68)^.

En el presente estudio de revisión, seis artículos presentan estrategias de prevención dirigidas únicamente a grupos de sexo masculino. La evidencia procedente de una revisión sistemática indica que los programas de prevención dirigidos a hombres y niños presentan resultados prometedores en la modificación de actitudes, creencias y normas sociales relacionadas con la violencia^(69)^. Una hipótesis planteada en la literatura es que los hombres se muestran más receptivos y cómodos con el contenido de los entrenamientos para espectadores y se sienten menos juzgados por sus compañeros cuando están en grupos con participantes del mismo sexo^(70)^. Cabe destacar la construcción social de la masculinidad en las diversas instituciones sociales, que da lugar a dinámicas de poder de los hombres sobre las mujeres por influencia del patriarcado, siendo necesario abandonar los privilegios que se les atribuyen en función del género. Por ello, los hombres pueden tener la percepción de que tienen algo que perder con la reestructuración social de los sistemas de poder^(71)^.

Es importante planificar estrategias para aumentar la participación masculina en programas destinados a prevenir la violencia de género en las universidades, ya que los hombres son los principales perpetradores de violencia contra las mujeres, incluso en las instituciones educativas. Un estudio de revisión integrativa sobre la prevalencia y los factores asociados a la violencia de género en la universidad identificó que la mayor parte de la violencia que sufren las mujeres es perpetrada por hombres, destacando las parejas íntimas, novios o compañeros^(72)^. Además, un estudio realizado con universitarios en EE. UU. identificó que entre el 20 % y el 26 % de ellos reportaron cierta propensión a perpetrar violencia, y la aceptación de ciertos mitos sobre la violación se asoció positivamente con dicha propensión^(73)^.

Asimismo, en los resultados se identificó el predominio de estrategias dirigidas a grupos mixtos de género, además de aquellas dirigidas a grupos exclusivamente de hombres o exclusivamente de mujeres. Por lo tanto, planificar actividades y capacitaciones complementarias diseñadas específicamente para cada grupo podría revelar el potencial de cada una de estas estrategias. Se deduce que, cuando el grupo es mixto, se puede propiciar el cultivo de relaciones colectivas y colaborativas. Además, planificar actividades exclusivas para hombres puede ser una forma de atraerlos y una oportunidad para abordar la influencia del patriarcado, buscando la responsabilidad social por el problema. Además, las actividades exclusivas para mujeres pueden ser más acogedoras e íntimas para intercambiar experiencias sobre vivencias de violencia.

En esta revisión de alcance, la capacitación de espectadores está destinada principalmente a la prevención de la violencia sexual; se han adoptado esfuerzos para prevenir la violencia sexual en la universidad en varias partes del mundo, aunque los estudios recuperados se han realizado principalmente en EE. UU. En América Latina, existe una creciente preocupación en las universidades por el desarrollo de protocolos para prevenir este tipo de violencia^(74)^.

Además, una revisión tras una búsqueda en Google Académico relacionó las principales palabras clave con el idioma de publicación de los artículos. En los artículos publicados en español, la categoría «violencia de género» fue la más frecuente. En esta categoría se pueden incluir diferentes formas de violencia, desde categorías genéricas, como «violencia contra la mujer», hasta otras más específicas, como violencia sexual, física, acoso, abuso, etc. En los estudios publicados en inglés, destaca el predominio de la violencia sexual, siendo una de las formas de violencia más estudiadas en las instituciones de educación superior de los países anglófonos^(75)^. Esto es compatible con los datos de la presente revisión en relación con el predominio de artículos realizados en EE. UU. sobre estrategias para prevenir la violencia sexual.

Sin embargo, el enfoque de un solo tipo de violencia puede ser una limitación, considerando especialmente la superposición de los tipos de violencia. La violencia sexual puede ocurrir tanto en el marco de una relación amorosa como en otros contextos (*p. ej.*, cuando el agresor es un desconocido, un conocido o un amigo)^(70)^. Además, la violencia de género se entiende como un fenómeno social derivado de las relaciones patriarcales, anclado en una estructura de poder desigual de los hombres sobre las mujeres, que opera en todos los ámbitos de la sociedad^(76)^, incluidas las universidades.

Este entrelazamiento de la violencia de género con la subordinación social de las mujeres no puede entenderse ni repararse de manera aislada del complejo más amplio de la violencia en la sociedad capitalista. Por lo tanto, es necesario articular luchas y acciones para desmantelar las relaciones sociales y las instituciones que oprimen a las mujeres^(77)^. Esto indica la necesidad de concebir programas de prevención que aborden la violencia de manera más integral, tratando más de un tipo de violencia en el mismo programa, teniendo en cuenta el contexto de las relaciones sociales entre los estudiantes universitarios.

Los hallazgos sobre los diversos tipos de programas de prevención concuerdan con los resultados de un metaanálisis sobre programas para prevenir la violencia en encuentros universitarios. En ese análisis, las estrategias utilizaban algún tipo de participación activa (77 %), como discusiones en grupo (55 %),presentaciones didácticas e interactivas (39 %); había otras actividades en grupo (36 %) y utilizaban algún componente en línea (29 %)^(70)^.

Discutir en grupo puede ser una forma de conocer y acercarse a los compañeros, aumentando el sentido de responsabilidad sobre el problema y la comprensión de que la violencia de género es un problema social en las universidades. Los resultados de un estudio cualitativo sobre violencia sexual en Guatemala mostraron que los estudiantes se sienten más seguros cuando se identifican y actúan juntos como grupo. La acción colectiva les indica a las posibles víctimas que no están solas, sino que cuentan con una red de apoyo, además de mostrar a los posibles agresores que sus acciones no serán toleradas^(78)^. Las sesiones grupales pueden estimular el diálogo y la participación de los estudiantes en las actividades. Las condiciones para desarrollar el pensamiento crítico se crean a través del diálogo, que implica el movimiento dialéctico entre el hacer y el pensar sobre el hacer, en un movimiento que amplía la conciencia^(79)^.

Además, la presente revisión identificó dos estrategias basadas en juegos. Una revisión sistemática presentó el uso de juegos como herramienta de aprendizaje entre estudiantes de enfermería. Permitió observar que el deseo de ganar la partida incentivó la participación y le dio realismo a la actividad, promoviendo un mayor compromiso y diversión, haciendo así que el aprendizaje fuera más placentero^(80)^.

En cuanto a la forma de implementación de los programas identificados en la presente revisión, la mayoría se llevó a cabo de manera presencial. Esto concuerda con los resultados de una revisión de alcance que analizó estrategias de prevención específicas sobre la violencia de pareja entre estudiantes universitarios; las intervenciones administradas en persona se examinaron en el 80 % de los estudios incluidos^(81)^. El aspecto social de la actividad presencial puede ayudar a los estudiantes a involucrarse más con el contenido de los programas, además de aportar una sensación de importancia al tema abordado, ya que los estudiantes pueden percibir la programación en línea como algo abstracto e impersonal. Los estudiantes también percibieron que la independencia que facilitan los enfoques en línea perjudica la eficacia del programa^(68)^.

En cuanto a la forma de ser impartidos, los resultados de un metaanálisis también señalaron que los métodos digitales, como los videos o los formatos exclusivamente en línea, son menos eficaces para desarrollar competencias relacionadas con el papel de espectador. Sugieren que la interacción presencial en un entorno colectivo es más eficiente para alcanzar este objetivo^(70)^.

La presencia de facilitadores se identificó en 20 de las 41 estrategias incluidas, y se localizó únicamente en las estrategias presenciales. Este dato es ligeramente inferior al reportado en un metaanálisis sobre programas para prevenir la violencia en encuentros universitarios, en el que el 61 % de las estrategias contaban con facilitadores^(70)^. Un metaanálisis sobre la eficacia de los programas para espectadores destinados a prevenir la violencia sexual mostró que la diferencia entre los efectos de los programas dirigidos por facilitadores y los autodirigidos, como videos o campañas de carteles, no fue significativa. Ambos formatos de programas tuvieron efectos positivos en las actitudes, creencias y comportamientos de los espectadores. Además, los efectos en los programas impartidos por un facilitador no variaron independientemente de si este era o no un compañero de la universidad^(67)^.

En la presente revisión, la duración y el número de sesiones de las estrategias variaron según su formato, lo que dificultó la síntesis de los datos. Sin embargo, se observó un predominio de sesiones únicas, con una duración que variaba entre minutos y horas para la mayoría de las estrategias (excepto las campañas de *marketing*, que tenían un mayor tiempo de exposición). Esto se repitió en el metaanálisis sobre programas para prevenir la violencia en encuentros universitarios, donde el 77 % de las estrategias se impartieron en una sola sesión, y las intervenciones duraron de 18 min a 12 h (la mayoría de ellas fue <2 h)^(70)^.

Otro estudio de revisión mostró que las intervenciones presenciales requieren más tiempo que las en línea para que los participantes las completen. Ese mismo estudio señaló la dificultad de la evaluación crítica para establecer estándares sobre el tiempo, el modo y la frecuencia de las intervenciones, ya que muchos estudios primarios no informaron datos esenciales sobre la implementación de la intervención^(81)^. Cabe, pues, considerar la evidencia de que la duración del programa se asoció positivamente con sus efectos en las actitudes y creencias de los espectadores, es decir, cuanto más largo el programa, mayor el efecto en las actitudes y creencias de los espectadores. Sin embargo, esta asociación no se produjo en el comportamiento de los espectadores^(67)^.

Aunque esta revisión de alcance no tiene como objetivo evaluar los efectos de las intervenciones, estos hallazgos permiten cuestionar su duración y continuidad. En este sentido, las universidades pueden beneficiarse de la implementación de programas más extensos o de acciones preventivas continuas, incluyendo enfoques multicomponentes. Además, la integración de las capacitaciones para espectadores en los planes de estudio de los cursos^(82)^, junto con la formación continua basada en evidencia científica para la comunidad^(83)^, son estrategias indicadas para construir un ambiente universitario seguro y justo.

Así, se destaca la importancia de los contenidos abordados en las capacitaciones. La presente revisión de alcance identificó el predominio de temas relacionados con actitudes, competencias e intervenciones de los espectadores; también se identificaron temas como los mitos relacionados con la violación y los estereotipos de género; definiciones, conceptos y epidemiología de la violencia; y el consentimiento y la comunicación saludable. Los temas relacionados con valores, conciencia, ética personal, recursos disponibles, servicios, riesgos, barreras y obstáculos fueron menos comunes.

Estos resultados concuerdan con los de otros estudios: las revisiones de los contenidos más comunes para las estrategias se centraron en el desarrollo de competencias (81 %) y la formación de espectadores (74 %), seguidas de discusiones sobre tipos de comportamientos violentos (42 %), la prevalencia de la violencia en el noviazgo y/o sexual (39 %), la definición de consentimiento (39 %), los recursos disponibles en el *campus* (36 %) y las consecuencias de la violencia en el noviazgo y/o sexual (32 %)^(70)^.

Además, una revisión que investigó los programas de intervención de espectadores, centrados en prevenir la violencia sexual en las universidades, encontró los siguientes temas más destacados: comportamiento del espectador, probabilidad de intervenir, eficacia, confianza y voluntad de actuar, así como cambios en las actitudes, creencias y normas^(84)^. El predominio de contenidos orientados a las actitudes, competencias e intervenciones de los espectadores (así como los mitos relacionados con la violación y los estereotipos de género) puede entenderse mejor a partir de los propios objetivos de las estrategias de intervención centradas en el espectador, que tienen como fin capacitar a los estudiantes para que desempeñen un papel activo en la reducción de la violencia de género.

La segunda categoría de contenidos identificada con mayor frecuencia en los estudios se relacionó con definiciones, conceptos y epidemiología de la violencia. Algunas evidencias indican que los programas que incluyen datos sobre la prevalencia local de la violencia produjeron mayores efectos en el sentimiento de eficacia y en las intenciones de los espectadores en relación con la prevención de la violencia en el relacionamiento. El estudio también sugirió que el conocimiento de las altas tasas de violencia en el *campus* puede generar una mayor conciencia sobre la gravedad del problema y la necesidad de reducir la prevalencia, lo que fomenta la intervención de los participantes^(70)^. Esto indica que los programas para prevenir la violencia de género pueden beneficiarse de la inclusión de datos locales sobre la epidemiología de la violencia en su programación. Así, el enfoque de definiciones culturalmente contextualizadas contribuye a que los participantes puedan reconocer situaciones y comportamientos violentos.

La información sobre los recursos disponibles y los servicios para ayudar a los estudiantes en situaciones de violencia en la universidad solo se abordó en cinco estudios de esta revisión. Uno de los estudios se realizó con estudiantes de pregrado en tres universidades y mostró que casi la mitad de ellos desconocía los recursos disponibles en su *campus* ^(85)^. Esto indica una limitación en las estrategias de prevención, ya que la falta de información sobre los servicios de apoyo institucional puede restringir las respuestas de los espectadores ante situaciones de violencia.

Por otro lado, la evidencia indica que el conocimiento de los estudiantes sobre las políticas y procedimientos de la universidad relacionados con la agresión sexual se relacionó significativamente con sus intenciones de involucrarse en comportamientos y/o actitudes prosociales como espectadores^(86)^. Las universidades deben, por lo tanto, formalizar políticas institucionales de prevención de la violencia de género, promoviendo el acceso de los miembros de la comunidad académica a recursos para la defensa de sus derechos e información sobre procedimientos de seguridad en casos de violencia^(82)^.

Se identificaron contenidos sobre riesgos, barreras y obstáculos a la intervención solo en cuatro estudios. Sin embargo, un estudio sobre violencia doméstica y sexual identificó consecuencias adversas para los espectadores que intervinieron en incidentes, incluyendo denuncias de acoso, amenazas y agresiones físicas^(87)^. Las estadísticas detalladas sobre la incidencia de diferentes tipos de violencia de género y la demografía de agresores y víctimas permiten adaptar los programas a las necesidades específicas de la comunidad universitaria. Además, la recolección y el análisis continuos de datos son fundamentales para evaluar la eficacia de las intervenciones, centrándose en las especificidades y los tipos de violencia, y realizando los ajustes necesarios. Esto implica analizar críticamente las estructuras sociales y económicas que sustentan la violencia, así como promover intervenciones que busquen transformar esas estructuras y crear un entorno académico más justo y seguro para todos.

Una limitación de este estudio fue la imposibilidad de acceder a todos los datos sobre la caracterización de las estrategias. No fue posible identificar la duración de las estrategias en uno de los artículos ni el número de sesiones en otros. Los objetivos específicos de cada capacitación no se sistematizaron.

## CONCLUSIÓN

Las capacitaciones para espectadores destinadas a prevenir la violencia de género en estudiantes universitarias se concentran mayoritariamente en contextos norteamericanos, con un enfoque predominante en el tema de la prevención de la violencia sexual. Existe una diversidad de formatos de intervención, que incluyen sesiones grupales, campañas de *marketing*, cursos en línea, talleres y juegos, con énfasis en acciones presenciales y grupos mixtos en cuanto al género. La mayoría de las estrategias tienen un carácter puntual, sin integración en las políticas institucionales. En las capacitaciones, los contenidos más abordados se centran en definiciones y conceptos de violencia, mitos y estereotipos de género, consentimiento y actitudes de los espectadores. Hay una exploración limitada de las barreras a la intervención y de los recursos institucionales en los contenidos de las estrategias, lo que puede limitar el alcance de las acciones de prevención de la violencia de género, lo que representa una laguna en las estrategias identificadas.

Este estudio señala la necesidad de intervenciones más amplias, que aborden diferentes formas de violencia de género y se realicen de manera continua. Las futuras intervenciones deben ampliar el alcance de los contenidos, incluyendo riesgos, barreras y recursos institucionales, y deben incorporarse a políticas permanentes de prevención en las universidades. Estas medidas contribuyen a construir entornos académicos más seguros, equitativos y comprometidos con la prevención de la violencia de género.

## Data Availability

Las estrategias de búsqueda utilizadas en cada fuente de información están disponibles en el siguiente *enlace*: (https://osf.io/x4pnf/files/hkvc6).
